# Growth phase matters: Boosting immunity via Lacticasebacillus‐derived membrane vesicles and their interactions with TLR2 pathways

**DOI:** 10.1002/jex2.169

**Published:** 2024-08-22

**Authors:** Miriam Sandanusova, Kristyna Turkova, Eva Pechackova, Jan Kotoucek, Pavel Roudnicky, Martin Sindelar, Lukas Kubala, Gabriela Ambrozova

**Affiliations:** ^1^ Faculty of Science, Department of Experimental Biology Masaryk University Brno Czech Republic; ^2^ Department of Biophysics of Immune System Institute of Biophysics of the Czech Academy of Sciences Brno Czech Republic; ^3^ Faculty of Science, Department of Biochemistry Masaryk University Brno Czech Republic; ^4^ Department of Pharmacology and Toxicology Veterinary Research Institute Brno Czech Republic; ^5^ Central European Institute of Technology (CEITEC) Masaryk University Brno Czech Republic

**Keywords:** growth curve, immunomodulation, *Lacticaseibacillus rhamnosus*, lipoteichoic acid, membrane vesicles, nanocarriers, TLR2

## Abstract

Lipid bi‐layered particles known as membrane vesicles (MVs), produced by Gram‐positive bacteria are a communication tool throughout the entire bacterial growth. However, the MVs characteristics may vary across all stages of maternal culture growth, leading to inconsistencies in MVs research. This, in turn, hinders their employment as nanocarriers, vaccines and other medical applications. In this study, we aimed to comprehensively characterize MVs derived from *Lacticaseibacillus rhamnosus* CCM7091 isolated at different growth stages: early exponential (6 h, MV6), late exponential (12 h, MV12) and late stationary phase (48 h, MV48). We observed significant differences in protein content between MV6 and MV48 (data are available via ProteomeXchange with identifier PXD041580), likely contributing to their different immunomodulatory capacities. In vitro analysis demonstrated that MV48 uptake rate by epithelial Caco‐2 cells is significantly higher and they stimulate an immune response in murine macrophages RAW 264.7 (elevated production of TNFα, IL‐6, IL‐10, NO). This correlated with increased expression of lipoteichoic acid (LTA) and enhanced TLR2 signalling in MV48, suggesting that LTA contributes to the immunomodulation. In conclusion, we showed that *Lacticaseibacillus rhamnosus* CCM7091‐derived MVs from the late stationary phase boost the immune response the most effectively, which pre‐destines them for therapeutical application as nanocarriers.

## Graphical abstract

Membrane vesicles (MVs) derived from Gram‐positive bacteria are crucial for bacterial communication, but their variable characteristics present challenges for applications as nanocarriers or vaccines. This study on *Lacticaseibacillus rhamnosus CCM7091‐*derived MVs, isolated throughout different growth phases, reveals significant protein differences, heightened uptake by Caco‐2 cells and immune stimulation in RAW 264.7 macrophages. Late stationary phase MVs (MV48) stand out, showing elevated lipoteichoic acid and enhanced TLR2 signalling, suggesting their potential as effective immunoboosting nanocarriers for future therapeutic applications.

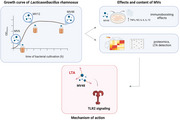

## 1 INTRODUCTION

Extracellular vesicles (EVs) are bi‐layered lipid membranous particles secreted by both eukaryotic and prokaryotic cells. Containing biological compounds such as nucleic acids, metabolites, lipids, polysaccharides and proteins (Gonzalez‐Lozano et al., 2022), EVs are considered to be an essential tool in cell‐to‐cell interaction and intercellular communication (Nahui Palomino et al., 2021). When localized inside the vesicle, the bioactive molecules are protected from environmental conditions and can be transferred to near or distant sites to exert their functions. This has great potential in clinics, as the EVs can be used as carriers for drug delivery and vaccination.

Bacterial EVs come with great opportunities and many advantages in medical applications when compared with their maternal bacteria, such as higher safety, inability to replicate and possible fusion with the target cell when delivering the cargo, while keeping the capacity to boost the immune system. They were first shown to be released by Gram‐negative bacteria more than 50 years ago (Chatterjee & Das, 1967) and were named outer membrane vesicles (OMVs). OMVs are already in use in clinics for vaccination (Micoli & MacLennan, 2020), however, their use is limited, because they can carry large quantities of lipopolysaccharide (LPS) causing too strong immune response (Park et al., 2021) and reduction of endotoxin activity is thus essential for the application (van der Ley & van den Dobbelsteen, 2011). The ability of Gram‐positive bacteria to produce EVs was long questioned because the thick peptidoglycan layer in the bacterial cell wall was thought to inhibit EV formation. The first direct evidence of the existence of EVs derived from Gram‐positive bacteria was provided by Lee et al. (2009). Since then, many genera of Gram‐positive bacteria were confirmed as producers of EVs, including *Bacillus* (Rubio et al., 2020), *Streptococcus* (Mehanny et al., 2022; Yerneni et al., 2021), *Clostridium* (Ma et al., 2022), *Enterococcus* (Wagner et al., 2018), *Lacticaseibacillus* (Choi et al., 2020; Tong et al., 2021), *Lactiplantibacillus* (Kurata et al., 2022) and others. In Gram‐positive bacteria, EVs are called membrane vesicles (MVs) and their size is approximately 20–500 nm in diameter (Champagne‐Jorgensen et al., 2021; Zavan et al., 2019). In the medical context, the MVs derived from Gram‐positive generally recognized as safe (GRAS) bacterial species such as *Lacticaseibacillus rhamnosus* emerge as even more tempting solution than the OMVs, due to their general safety and absence of endotoxins (Chen et al., 2022; Kim et al., 2017).

*Lacticaseibacillus rhamnosus* is a facultative anaerobic Gram‐positive bacterium, isolated from a variety of habitats, including the human intestinal tract. Certain strains are known for their beneficial and immunomodulatory properties (Zheng et al., 2020). The effects of *L. rhamnosus‐*derived MVs may be similarly useful, as they may reflect the contents of the maternal cells, which was already proven in many studies (Al‐Nedawi et al., 2015; Behzadi et al., 2017; Champagne‐Jorgensen et al., 2021; Gu et al., 2021; He et al., 2017; Keyhani et al., 2022; Mata Forsberg et al., 2019; Tong et al., 2021). Nevertheless, the proteome, transcriptome and functional properties of the culture of *L. rhamnosus* change depend on the cultivation conditions (Koskenniemi et al., 2009), growth phase (Laakso et al., 2011) or exposition to environmental stress (Aakko et al., 2014). Therefore, the information contained in the MVs can be altered accordingly (Kim et al., 2019; Mehanny et al., 2022; Tashiro et al., 2010; Wang et al., 2021). Up to date, to our best knowledge, no studies clarify how the different culture conditions influence the MVs uptake, interaction and recognition by the recipient cells (either epithelial or immune).

The lipoteichoic acid (LTA), Gram‐positive bacteria membrane glycolipid that has a diacylated glycerol group attached to a sugar backbone and repeating units (Kang et al., 2009), is one of the pathogen‐associated molecular patterns (PAMPs) recognized by the immune system of the host. LTA stimulates immune reaction through Toll‐like receptor 2 (TLR2) (Knapp et al., 2008; Long et al., 2009; Nilsen et al., 2008), which in turn activates the NF‐κB pathway and pro‐inflammatory mediators production (Kawai & Akira, 2007). LTA localization changes during the growth of several strains of *Lacticaseibacilli* (Shiraishi et al., 2018). As for their MVs, the presence of LTA in MVs was already confirmed (Champagne‐Jorgensen et al., 2021; Uppu et al., 2023), although to our best knowledge, a study comparing LTA expression in MVs at different time points is lacking. Throughout the relatively short history of MV research, the MVs have often been isolated and characterized at only one selected time point of the growth phase (Choi et al., 2022; Han et al., 2023; Keyhani et al., 2022; Kurata et al., 2022). Only in the last 2 years have studies begun to compare MVs from at least two phases of maternal bacteria growth. For instance, da Luz et al. reported that the biochemical cargo of *Staphylococcus aureus*‐derived MVs changed depending on the growth phase of the mother cells (da Luz et al., 2022). The time of the harvest influenced also *Streptococcus pneumonia*‐derived MVs’ amount and differences were found in their interaction with primary macrophages (Mehanny et al., 2022).

Still, to the best of our knowledge, a comprehensive study covering the entire growth curve of the maternal bacteria and its influence on the composition and function of MVs is lacking. We believe that for using the entire potential of MVs in clinical applications (such as vaccine development and nanocarriers for drug delivery), it is of great importance to characterize and standardize the optimal time‐point of the MVs isolation, with regards to their immunoboosting properties, maximal yields and absolute reproducibility. Here, we aimed to describe the impact of the growth phase of the microbial culture of *L. rhamnosus* CCM7091 on the biophysical characterization, uptake by the intestinal cells, immunomodulatory properties and content of its MVs. We hypothesized that the biophysical and biochemical composition of *Lacticaseibacillus rhamnosus*‐derived MVs changes during the entire growth curve and thus the immunoboosting properties of these MVs may be different.

## 2 MATERIALS AND METHODS

We have submitted all relevant data from our experiments to the EV‐TRACK knowledgebase (EV‐TRACK ID: EV230796) (EV‐TRACK Consortium et al., 2017).

### 2.1 Bacterial strain and growth conditions

The bacterial strain *Lacticaseibacillus rhamnosus* CCM 7091 (=ATCC 53103) was obtained from the Czech Collection of Microorganisms (CCM, Brno, Czech Republic) and cultivated aerobically in de Man, Rogosa and Sharpe broth (MRS) medium (VWR, Czech Republic) at 37°C for 48 h. The bacterial growth was determined by measuring the optical density at 600 nm in a 96‐well plate (OD_600nm_, Infinite M200 iControl, Tecan, Switzerland) and by plating on MRS agar medium prepared by adding 1.5% agar (VWR, Czech Republic). The colonies were counted after 48 h of incubation at 37°C (CFU mL^−1^).

### 2.2 Isolation and purification of MVs

*Lacticaseibacillus rhamnosus* CCM7091 was grown in 0.22‐µm‐filtered MRS medium at 37°C for 24 h under aerobic conditions without shaking. The overnight culture (OD_600nm _= 1.5) was sub‐cultured at a ratio of 1:100 into the final volume of 500‐mL MRS broth, followed by incubation at 37°C. Three cultivation end‐time points of the growth curve were selected: the early exponential growth phase (6 h, MV6), the late exponential growth phase (12 h, MV12) and the late stationary phase (48 h, MV48). At the appropriate end‐time point, the bacterial cell culture was centrifuged at 8800 × *g* for 30 min at 4°C to separate bacterial cells and unwanted debris (MR22i Refrigerated Centrifuge, Jouan, USA), and OD_600nm_ and CFU mL^−1^ were determined. The supernatant was filtered using a 0.45‐µm pore membrane filter (PVDF, Whatman, Cyntiva, Germany) to remove any remaining bacterial cells. The filtrate was checked for sterility by plating 100 µL of filtrate on MRS agar and cultivated for 48 h at 37°C. The filtrate was ultracentrifuged at 175,000 × *g* for 2 h (32,000 rpm for rotor SWTi32; Optima TM LE‐80K Preparative Ultracentrifuge, Beckman Coulter, USA). After the ultracentrifugation, the supernatant was discarded, and the pellet was re‐suspended in sterile 0.22‐µm‐filtered phosphate‐buffered saline (PBS; pH 7.5). Of note, in all the experiments when the PBS was needed, the sterile 0.22‐µm‐filtered PBS (pH 7.5) was used. The crude fraction of MVs was then purified using a sucrose cushion as described by Pospichalova et al. (2015) with slight modifications. Briefly, the crude fraction was re‐suspended in PBS, underlaid with 30% sucrose solution (30% sucrose in 20‐mM Tris, pH 7.6 in D_2_O, 0.22‐µm‐filter sterilized) and ultracentrifuged at 175,000 × *g* for 2 h at 4°C (32,000 rpm for rotor SWTi32; slow deceleration; Optima TM LE‐80K Preparative Ultracentrifuge, Beckman Coulter; USA). The interface was collected, refilled with PBS, ultracentrifuged at 175,000 × *g* for 2 h at 4°C (32,000 rpm for rotor SWTi32; Optima TM LE‐80K Preparative Ultracentrifuge, Beckman Coulter, USA) and the pellet was re‐suspended in ±100 µL of PBS to obtain the final purified MVs fraction (Figure S1). The MVs were stored at −80°C until use.

### 2.3 Size and particle concentration analysis

The Multi‐angle Dynamic Light Scattering (MADLS) technique was used for the size and particle concentration analysis. Measurements were performed using Zetasizer Ultra (Malvern Panalytical Ltd, UK) equipped with a He–Ne laser at a wavelength of 633 nm at a constant temperature of 25°C. The light scattering data were collected at three angles, 173°, 90° and 13°, and evaluated using ZS Xplorer software version 3.20 (Malvern Panalytical Ltd, UK). Results for the hydrodynamic diameter, polydispersity index (PDI) and particle concentration are reported as the mean value of four independent measurements ± standard deviation (SD). The difference in particle size between samples was determined using a one‐way analysis of variance (ANOVA) followed by Tukey's honestly significant difference (HSD) test. Statistical analysis was performed using GraphPad Prism 8 (GraphPad Software, San Diego, California, USA).

### 2.4 Visualization by electron microscopy

Four microlitres of the MVs sample were applied to freshly plasma‐cleaned TEM grids (Quantifoil, Cu, 300mesh, R2/1) and vitrified in liquid ethane using Leica Automatic Plunge Freezer EM GP2 (4°C, 100% rel. humidity, 30‐s waiting time, 4‐s blotting time and using sensor blotting). The grids were subsequently mounted into the Autogrid cartridges and loaded into Talos Arctica (Thermo Fisher Scientific, USA) transmission electron microscope for imaging. The microscope was operated at 200 kV. The Cryo‐TEM micrographs of MVs were collected on the K2 direct electron detection camera at a nominal magnification of 79,000× with an average defocus of −2.2 µm and an overall dose of ∼40 eÅ^−2^.

### 2.5 Fluorescent labelling of the MVs and their uptake by intestinal cells detection

The MVs were diluted to a concentration of 10^12^ particles per mL in labelling buffer (50‐mM Na_2_CO_3_, 100‐mM NaCl, pH 9.2) and Octadecyl Rhodamine B Chloride (Biotium, USA) (R18) was used as the fluorescent probe. R18 was added to the MVs in a final concentration of 1.5 µg mL^−1^ and incubated for 30 min at 25°C with shaking. Subsequently, 3.5 mL of PBS was added and the suspension was underlaid with 1 mL of 30% sucrose solution (30% sucrose in 20‐mM Tris, pH 7.6 in D_2_O, 0.22‐µm‐filter sterilized) and ultracentrifuged at 175,000 × *g* for 2 h at 4°C (38,000 rpm for rotor SW55Ti; slow deceleration; Optima TM LE‐80K Preparative Ultracentrifuge, Beckman Coulter; USA). The interface was collected, refilled with PBS to wash the free label and ultracentrifuged at 175,000 × *g* for 2 h at 4°C (38,000 rpm for rotor SW55Ti; slow deceleration; Optima TM LE‐80K Preparative Ultracentrifuge, Beckman Coulter; USA). The R18‐labelled MVs were re‐suspended in 100 µL of PBS and immediately used for Caco‐2 cell treatment.

The human colorectal carcinoma cell line Caco‐2 (American Type Culture Collection, USA) was routinely cultured in Dulbecco's Modified Eagle Medium (DMEM; Gibco, USA), supplemented with 10% (vv^−1^) foetal bovine serum (FBS; Thermo Fisher Scientific, USA) and 100 U mL^−1^ of penicillin and 100 µg mL^−1^ of streptomycin (Gibco, USA) and 5‐mM pyruvate. Cells were cultivated at 37°C in a humidified 5% CO_2_ atmosphere. For the experiments, 2.0 × 10^4^ cells per well were seeded to a 96‐well plate and differentiated for 21 days. One day before the MV uptake experiment, the medium was replaced with a medium without antibiotics and FBS. To study the uptake mechanism, inhibitors of various MVs uptake pathways were employed: chlorpromazine (30 µM), genistein (10 µM), nystatin (10 µM) or wortmannin (1 µM) (all Sigma Aldrich, USA); 1 h prior to the MV48 addition. The R18‐labelled MVs were added to the cells in a concentration of 10^5^ particles per cell and the fluorescence was measured until it reached plateau (Infinite M200 iControl, Tecan, Switzerland) (λ_Ex_ = 560 nm; λ_Em_ = 590 nm). The time‐course internalization was assessed as previously described (Gnopo & Putnam, 2020). Briefly, the fusion efficiency of the uptake was determined using Equation (1), where *F_0_* represents the fluorescence of the labelled MVs in the absence of cells, *F_T_* is the increasing fluorescence over time, and *F_I_* corresponds to the fluorescence observed at infinite dilution when 60‐mM Triton X‐100 (Sigma Aldrich, USA) was added (Triton X‐100 being a detergent capable of dequenching all remaining R18, yielding a maximum fluorescence signal). Following these calculations, the resulting curve was fitted using Equation (2) to extract the kinetic parameters of the uptake. Specifically, *Y* represents the fusion efficiency at time *t*, *Y_max_* corresponds to the maximum fusion efficiency and *k* represents the fusion rate constant. Fluorescence assays were repeated in four to six independent measurements, in technical triplicates. The statistical analysis was conducted using a *t*‐test in R Studio.

(1) $$\%\;=\frac{F_{T}-F_{0}}{F_{I}-F_{0}}$$

(2) $$Y\;=Y_{max}\;\left(1-e^{-kt}\right)$$

### 2.6 Preparation of RAW 264.7 cell line and the treatment with MVs

The murine macrophage cell line RAW 264.7 (American Type Culture Collection, USA) was routinely cultured in DMEM (Gibco, USA), supplemented with 10% (vv^−1^) FBS (Thermo Fisher Scientific, USA), 100 U mL^−1^ of penicillin and 100 µg mL^−1^ of streptomycin (Gibco, USA). Cells were cultivated at 37°C in a humidified 5% CO_2_ atmosphere. To analyse the production of cytokines and reactive nitrogen species (RNS), the cells were seeded in a 24‐well plate at a density of 2.5 × 10^5^ cells per well. Twenty‐four hours after the seeding, the medium was replaced with a medium without antibiotics, and the cells were treated for 24 h with three concentrations of MVs: 1.0 × 10^7^, 1.0 × 10^5^ and 1.0 × 10^3^ MVs per cell. As a negative control, the vehicle alone (PBS) was used; whereas 100 ng mL^−1^ LPS (*Escherichia coli* O26:B6 LPS, cat. n. 297‐473‐0, Merck, Germany) was used as a positive control. The media were then collected, depleted of any remaining cells (by centrifugation, 250 × *g* for 5 min at 4°C), and stored at −20°C for further cytokine and RNS detection. The statistical analysis was performed using ANOVA followed by Dunnett's multiple comparisons test in R Studio; three independent repetitions were performed.

### 2.7 ELISA

Levels of the inflammatory cytokines TNFα and IL‐6, the immunoregulatory cytokine IL‐10 and IL‐8 were detected in the cell culture medium collected after the treatment of RAW 264.7 cells or HEK293 with the MVs. Detection was performed by ELISA (cat. n. 88‐7324‐88, 88‐7064‐88, 88‐7105‐88, 88‐8086‐88, Thermo Fisher Scientific, USA) following the manufacturer's instructions with no modifications.

### 2.8 Measurement of nitrite concentration by Griess reaction

Changes in RNS production were measured indirectly as the accumulation of nitrites, the end product of NO metabolism, in a medium using the Griess spectrophotometric assay, as previously described (Arias‐Negrete et al., 2004). At the end of the incubation period, the culture media were collected from the wells and centrifuged at 250 × *g* at 4°C for 5 min. Then 80 µL of supernatant was mixed with an equal volume of Griess reagent (Sigma Aldrich, USA) in a 96‐well plate and the mixture was incubated at room temperature and in the dark for 30 min. Absorbance was measured at 546 nm (Spectrophotometer Sunrise, Tecan, Switzerland). Sodium nitrite was used as a standard.

### 2.9 Proteomic sample preparation and analysis by liquid chromatography–tandem mass spectrometry

Proteins extracted from MVs of *Lacticaseibacillus rhamnosus* CCM 7091 isolated at defined time points of the growth curve (6, 12 and 48 h) were subjected to filter‐aided sample preparation as described elsewhere (Wisniewski et al., 2009). The resulting peptides were analysed by liquid chromatography–tandem mass spectrometry (LC–MS/MS) performed using an UltiMate 3000 RSLCnano system (Thermo Fisher Scientific, USA) coupled on‐line to a timsTOF Pro spectrometer (Bruker, USA). See the supplemental material section for full details of the analysis and data evaluation. The mass spectrometry proteomics data have been deposited at the proteomeXchange Consortium via the PRIDE (Perez‐Riverol et al., 2022) partner repository with the dataset identifier PXD041580.

### 2.10 Bioinformatic processing of proteomic outputs

Proteins that did not meet the condition of identification by at least two unique peptides were filtered out. Further, all proteins that were found in at least one sample from triplicates of MV6, MV12 and MV48 were selected for processing. Protein cell localizations were predicted using PSORTb software (Yu et al., 2010). The annotation and classification of proteins into functional groups were carried out by KEGG Mapper (Kanehisa, 2019; Kanehisa & Goto, 2000; Kanehisa et al., 2023). To visualize the protein overlap, Venn diagrams were generated using the ggVennDiagram package in R. A heatmap was generated using log_2_‐transformed and imputed intensities with the ComplexHeatmap package in R, and the default clustering method, hierarchical clustering, was used to group similar data. Principal component analysis (PCA) was performed on log_2_‐transformed and imputed intensities using the ggplot2 package in R. Volcano plots were generated using the VolcaNoseR web application (Goedhart & Luijsterburg, 2020). LIMMA‐calculated statistics and log_2_‐transformed fold changes were used to visualize the differential expression of proteins.

For the prediction of protein–protein interactions (PPI), the MV48 protein dataset was used, comprising proteins that were identified with at least two peptides in at least one replicate and exhibited at least a twofold increase in abundance compared to MV6 and MV12. The interactions were predicted with proteins from the mouse IL‐6, IL‐10 and TNFα signalling cascades and human TLR pathways, which were retrieved from the Reactome database (Milacic et al., 2024). The interolog prediction on the PredHPI Webserver (Loaiza & Kaundal, 2021) was employed to predict interactions, with the criteria set to a minimum coverage of 60% and a sequence identity threshold of 30% for each interacting partner.

### 2.11 Western blotting and immunodetection of LTA

The LTA levels in MV6, MV12 and MV48 were detected by Western blot analysis with immunodetection, as previously described (Ambrozova et al., 2010). Briefly, equal volumes of MV pellets were mixed with Leammli buffer and heated to 100°C for 10 min, then loaded and subjected to SDS‐polyacrylamide gel electrophoresis using 12.5% running gel. For reference, PageRuler Plus Prestained Protein Ladder (cat. n. 26619, Thermo Fisher Scientific, USA) was loaded. Anti‐LTA monoclonal antibody (cat. n. MA1‐7402, Thermo Fisher Scientific, USA) and anti‐mouse IgG horseradish peroxidase‐linked whole antibody (from the horse; Cell Signaling Technology, USA) were used. Immunoreactive bands were detected using an ECL™ detection reagent kit (Pierce, USA) by Amersham™ Imager 680 and quantified by scanning densitometry using the Image J™ programme. The individual band density value was expressed in arbitrary units. For statistical analysis, ANOVA followed by Dunnett's multiple comparison test in GraphPad Prism 6 (GraphPad Software, San Diego, California, USA) was used; three independent experiments were performed.

### 2.12 Toll‐like receptor (TLR) screening assay

HEK293 (ATCC, USA) transfectants TLR2/CD14, TLR6/2 and TLR1/2 were stably transfected with pDUO plasmids purchased from InvivoGen (USA) according to the manufacturer's instructions. pDUO‐hCD14/TLR2, pDUO‐hTLR1/TLR2 pDUO‐hTLR6/TLR2 are expression vectors designed to co‐express CD14 and TLR2 genes, TLR1 and TLR2 genes and TLR6 and TLR2 genes. Further, commercially available HEK293 cells stably transfected with the human TLR4a, MD2 and CD14 genes, NOD1 genes and NOD2 genes were used (293/hTLR4A‐MD2‐CD14, 293‐hNOD1 and 293‐hNOD2 cells) and HEK 293 control cell line (293/null cells) was tested as a scrambled control (InvivoGen, USA).

Transfected cells were grown in DMEM (Gibco, USA) supplemented with 10% (vv^−1^) FBS (Thermo Fisher Scientific, USA), 1% (vv^−1^) L‐glutamine (Gibco, USA) and 100 U mL^−1^ of penicillin and 100 µg mL^−1^ of streptomycin (Gibco, USA) with selective antibiotic Blasticidin (10 µg mL^−1^; InvivoGen, USA) applied every 5th passage. For the experiment, cells were seeded in a medium without antibiotics at 2.0 × 10^4^ cells per well in 96‐well plates and treated for 24 h with MVs in concentration 10^5^ MVs per cell. Positive controls for each cell line were as follows: 10^8^ HKLM mL^−1^ for TLR2/CD14, 50 ng mL^−1^ Pam3CSK4 for TLR1/2, 10 ng mL^−1^ FSL1 for TLR6/TLR2, 10 mg mL^−1^ LPS for TLR4A‐MD2‐CD14, 100 µg mL^−1^ iE‐DAP for NOD1, 10‐ng mL^−1^ L18‐MDP for NOD2 (InvivoGen, USA) cells. Cell culture supernatants from four independent experiments were assayed for IL‐8 ELISA according to the manufacturer's instructions. Differences between samples were verified using R Studio and statistically analysed by ANOVA. Data normalization was performed using a *Z*‐score.

### 2.13 Statistical analysis

Data are presented as mean ± SD. All measurements were performed at least in duplicate in *n* independent experiments. The statistical significance was assessed at *p < 0.05. Details regarding statistical analyses can be found in the description of the particular methods.

## 3 RESULTS

### 3.1 *Lacticaseibacillus rhamnosus*‐derived MVs average size increases with a longer cultivation period

First, to detect changes in the growth of *Lacticaseibacillus rhamnosus* CCM7091 cells, the overnight culture was inoculated in MRS broth and incubated aerobically at 37°C for 48 h, and we determined OD_600nm_ and CFU mL^−1^ throughout the 48‐h incubation. According to the growth curve (Figure S2) and the CFU mL^−1^ (Table S1), we selected the time points for MV isolation to represent different growth phases: the early exponential phase (6 h, MV6), the late exponential phase (12 h, MV12) and the late stationary phase (48 h, MV48).

**FIGURE 1:**
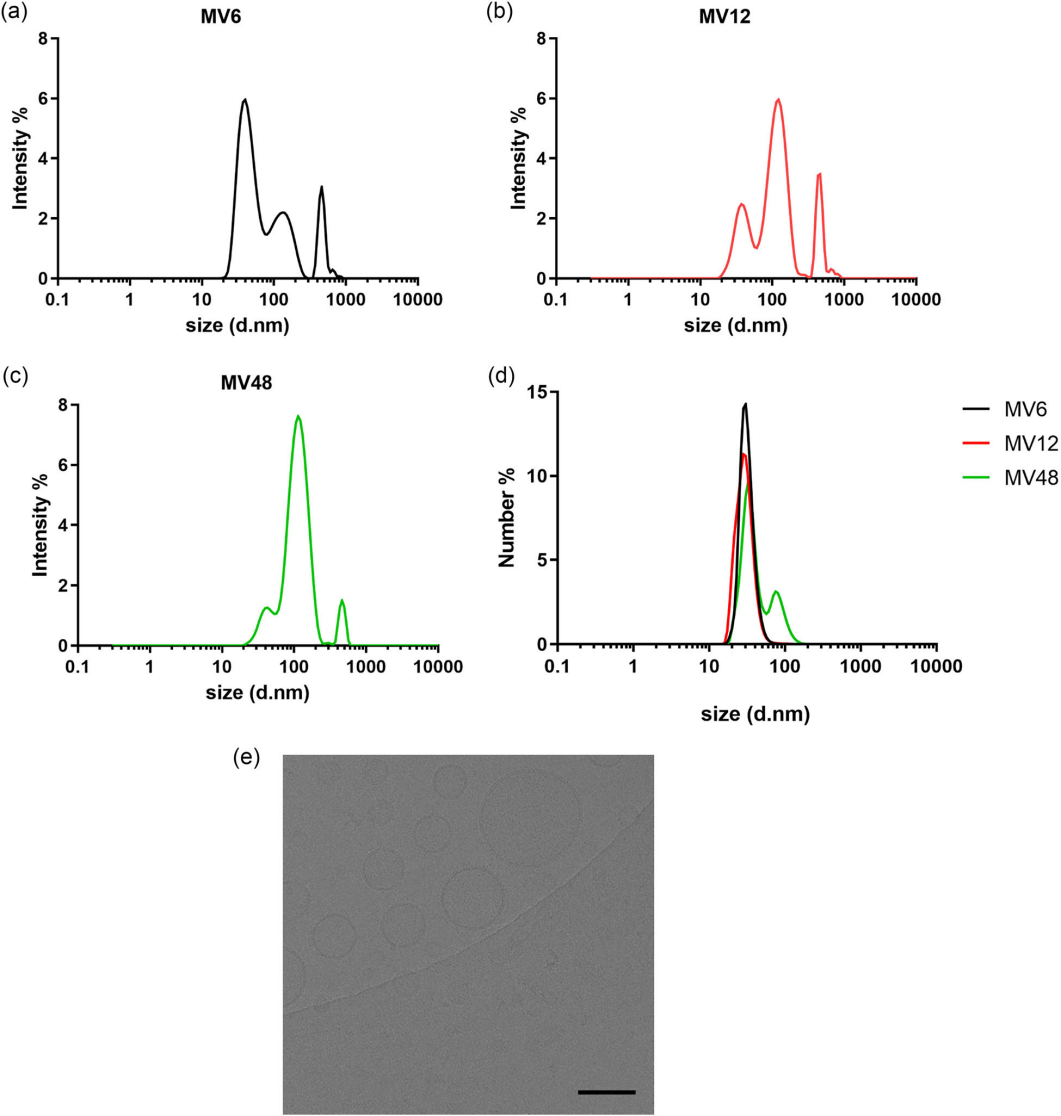
Biophysical characterization of *Lacticaseibacillus rhamnosus* CCM7091 membrane vesicles (MVs) isolated throughout bacterial growth (after 6, 12 and 48 h – MV6, MV12, MV48). (a)–(c) Size distribution by intensity (MV6, MV12 and MV48, respectively). (d) Size distribution by number. (e) Cryo‐electron microscopy visualization of MV48 (bar length is 100 nm).

After harvesting and purifying the MVs at three‐time points of the growth curve, we performed biophysical characterization using MADLS. Generally, the *L. rhamnosus* CCM7091‐derived MVs consisted of two main populations with peak maxima around 30 nm for the first population and around 100 nm in diameter for the second population, as shown in intensity distribution analysis (Figures 1a–c and S3). The third population with a peak maximum of around 500 nm (Figure 1a–c) represents potential impurities or aggregates in the sample, which were nevertheless in the minority as can be seen in the Number distribution analysis (Figure 1d). When comparing the particle size across the batches of MVs isolated at the three time points, the average particle diameter was 72 ± 14, 95 ± 10 and 101 ± 7 nm for MV6, MV12 and MV48, respectively (Table 1), with the difference between MV6 and MV48 being statistically significant. This can be explained by a larger proportion of the smaller population around 30 nm in the MV6 sample compared to the MV48, where the population around 100 nm has a higher contribution to the total concentration of particles in the sample. The presence of multiple particle populations in all selected samples is also reflected in the PDI. Generally, high values (approximately 0.2 and higher) of the PDI index indicate a broad size distribution. Nevertheless, compared to other populations, MV48 shows a homogeneous population character with an average PDI of 0.263 ± 0.070 compared to MV6 with a PDI of 0.369 ± 0.054. Next, we examined the morphology of *L. rhamnosus*‐derived MVs using cryo‐electron microscopy (cryo‐EM). The representative image shows that the MV48 sample consisted of lipid bi‐layer vesicles of varying sizes (Figure 1e).

**TABLE 1:** Size and concentration of *Lacticaseibacillus rhamnosus* CCM7091‐derived membrane vesicles (MVs) after cultivation for 6, 12 and 48 h (MV6, MV12, MV48), analysed by MADLS.

Name	*n*	*Z*‐average (nm)	Polydispersity index (PDI)	Concentration (particles mL^−1^)
MV6	1	80 ± 2	0.383 ± 0.015	1.80·10^13^
	2	91 ± 11	0.450 ± 0.062	7.40·10^12^
	3	56 ± 3	0.305 ± 0.006	1.40·10^12^
	4	60 ± 6	0.337 ± 0.026	3.80·10^12^
	Average	72 ± 14	0.369 ± 0.054	7.65 ± 6.35 ·10^12^
MV12	1	84 ± 2	0.408 ± 0.014	2.00·10^12^
	2	93 ± 3	0.447 ± 0.028	2.10·10^12^
	3	92 ± 0	0.245 ± 0.009	1.20·10^14^
	4	111 ± 2	0.616 ± 0.005	1.00·10^13^
	Average	95 ± 10	0.429 ± 0.132	3.35 ± 5.00 ·10^13^
MV48	1	103 ± 1	0.198 ± 0.010	5.00·10^13^
	2	88 ± 1	0.217 ± 0.005	5.30·10^12^
	3	106 ± 3	0.257 ± 0.017	9.93·10^12^
	4	105 ± 4	0.379 ± 0.006	1.40·10^12^
	Average	101 ± 7*	0.263 ± 0.070	1.67 ± 1.95 ·10^13^

*Note*: One‐way ANOVA was followed by Tukey's honestly significant difference (HSD) test. *n* = 4.

*p < 0.05; statistical significance for MV48 *Z*‐average versus MV6 *Z*‐average.

### 3.2 The uptake of late‐stationary‐phase *L. rhamnosus* MVs is the most efficient and the inhibitor of the caveolae‐mediated pathway is the most effective in blocking their uptake by Caco‐2 cells

Physiologically, the intestinal cells are the first cell type to encounter the gut microbiota and its EVs. For that reason, we have chosen the cell line Caco‐2 (colorectal carcinoma cell line commonly used as a model of the intestinal monolayer) to monitor the uptake of MVs by the intestinal epithelium. We stained the MVs by lipophilic probe R18, which fluorescence is quenched at high R18 concentrations, but de‐quenched when the probe is diluted. Then, we incubated the labelled MVs with Caco‐2 cells, spectrophotometrically detected the fluorescence plateau and calculated the kinetic parameters.

**FIGURE 2:**
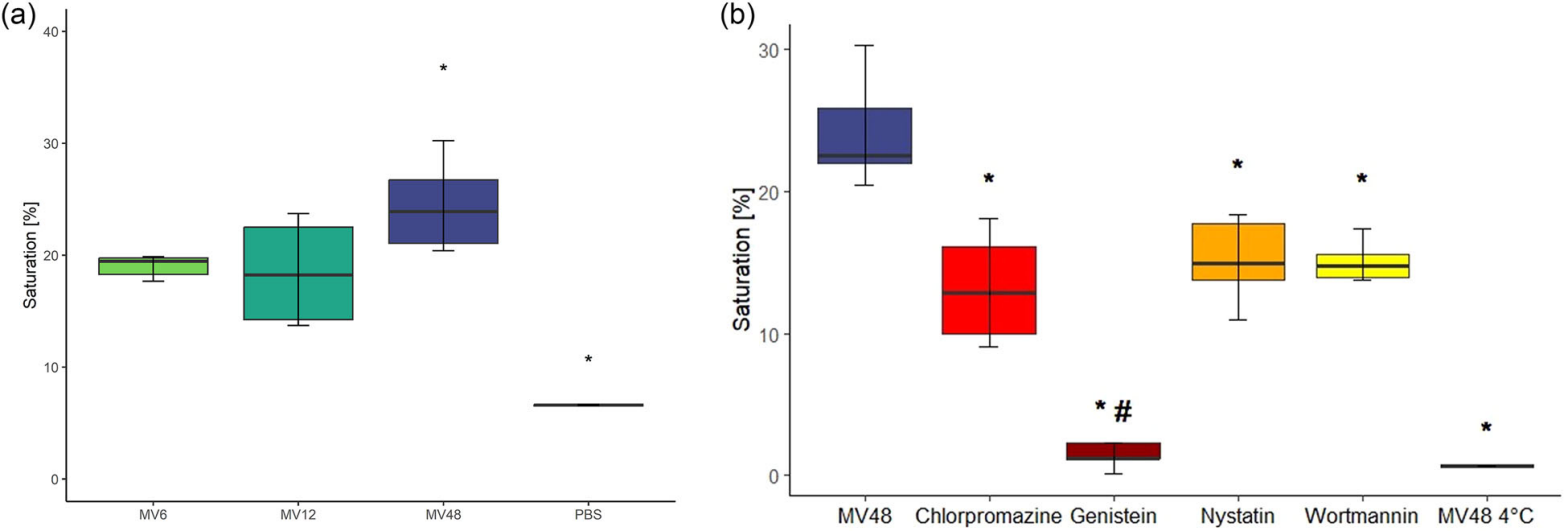
Uptake of *Lacticaseibacillus rhamnosus* CCM7091 membrane vesicles (MVs) by Caco‐2 cells. (a) The final saturation observed approximately after 10‐h incubation of R‐18 labelled MVs from different times of isolation: 6, 12 and 48 h (MV6, MV12, MV48) with Caco‐2 cell monolayer. The increase in fluorescence was monitored until saturation. The vehicle alone, subjected to the same labelling protocol, was used as negative control (PBS). (b) Determination of MV48 uptake pathways. Selective inhibitors targeting different uptake pathways were employed 1 h before MV48 treatment: chlorpromazine (red) for clathrin‐mediated endocytosis, genistein (dark red) for caveolin‐mediated endocytosis, nystatin (orange) for lipid rafts and wortmannin (yellow) for micropinocytosis. The uptake of MV48 in the absence of inhibitors is represented as a control (blue). Additionally, MV uptake was assessed at 4°C to demonstrate the energy dependence of the process. The box plots show medians (lines), interquartile ranges (boxes) and minimum and maximum values (whiskers); statistical analysis was performed using the *t*‐test and the significance was assessed at *p* < 0.05: * marks a statistically significant difference between MV48 without inhibitors versus Chlorpromazine, Genistein, Nystatin, Wortmannin, MV48 4°C; # marks a statistically significant difference between Genistein versus Chlorpromazine, Nystatin, Wortmannin; *n* = 4–6.

Our results indicate that MVs were effectively internalized by the Caco‐2 cell monolayer, as the fluorescence signal increased during the uptake (Figure 2a). The uptake of MV6 and MV12 reached a maximum of 19% by recipient cells, while the uptake of MV48 was significantly higher, reaching 25% (Figure 2a and Table 2). As a negative control, we subjected the vehicle alone (PBS) to the same labelling protocol as the MVs. Its internalization exhibited a different time‐course of internalization compared to that of labelled MVs (Figure S4).

**TABLE 2:** Kinetic parameters of *Lacticaseibacillus rhamnosus* CCM7091 membrane vesicles (MVs) uptake by Caco‐2 cells.

	MV6	MV12	MV48	PBS
Plateau [%]	18.83	18.28	24.32	6.600
K [h^−1^]	0.293	0.259	0.280	0.273
Half‐life [h]	2.364	2.677	2.478	2.537
Tau [h]	3.410	3.862	3.574	3.660

*Note*: Fluorescently labelled MVs isolated at different times of cultivation (MV6, MV12, MV48) were cultivated with Caco‐2 cells until uptake saturation was reached. The parameters encompass the saturation plateau, kinetic rate constant (*K*), half‐life (defined as the time required to attain half of the saturation level) and tau (signifying the time constant of saturation).

The uptake mechanism of the MVs may have several forms, of which the most probable for MVs uptake are endocytosis, micropinocytosis and lipid rafts‐mediated internalization. To identify the prevailing uptake mechanism of *L. rhamnosus* MVs by Caco‐2 cells, we tested the inhibitors of the chosen pathways: chlorpromazine, genistein, nystatin, wortmannin for clathrin‐ and caveolae‐mediated endocytosis, lipid rafts‐dependent and micropinocytosis, respectively (as nicely reviewed by O'Donoghue & Krachler (2016)). Treatment with all the inhibitors resulted in a statistically significant decrease in MV48 uptake. Genistein was the most effective in the uptake inhibition, reducing the saturation significantly compared with the rest of the inhibitors (Figure 2b and Table 3). Therefore, the MV uptake mechanism by Caco‐2 cells is likely a combination of endocytosis, micropinocytosis and lipid rafts‐dependent uptake, and the predominant pathway is the caveolae‐mediated endocytosis.

**TABLE 3:** Kinetic parameters of *Lacticaseibacillus rhamnosus* CCM7091‐derived membrane vesicles (MVs) uptake by Caco‐2 cells treated with selective inhibitors of uptake pathways.

	MV48	Chlorpromazine	Genistein	Nystatin	Wortmannin	MV48 4°C
Plateau [%]	24.32	14.76	1.47	15.59	15.49	0.70
K [h^−1^]	0.280	0.478	0.297	0.581	0.573	1.688
Half‐life [h]	2.478	1.450	2.333	1.193	1.210	0.411
Tau [h]	3.574	2.092	3.366	1.722	1.745	0.592

*Note*: The MVs were obtained after 48‐h bacterial cultivation (MV48). The Caco‐2 cells were treated with the inhibitors (chlorpromazine, genistein, nystatin or wortmannin) for 1 h before the addition of MV48, and kinetic parameters were calculated. The parameters encompass the saturation plateau, kinetic rate constant, half‐life (defined as the time required to attain half of the saturation level) and tau (signifying the time constant of saturation). As negative controls, the MV48 were added to Caco‐2 cells without the inhibitors (MV48), and the same was measured also at 4°C without the presence of inhibitors (MV48 4°C).

To exclude the possibility that the MV uptake is a passive process, we conducted the experiment also at 4°C, when the transcription, translation and metabolic activity of the recipient cells are reduced (Hunt et al., 2005). The resulting fusion efficiency, which we calculated, reached values up to 0.8% for all MVs (Figure 2b and Table S2), thus we can confidently conclude that the uptake of MVs is indeed an active, energy‐dependent process.

We further tested whether the MVs induce inflammation‐related activation of the Caco‐2 cells. We measured IL‐8 levels by ELISA, however, the MVs did not have any significant effects on IL‐8 production (data not shown).

### 3.3 *L. rhamnosus* MVs from the later stages of the growth curve exert immunomodulatory properties

It is known that in the intestine, the bacterial EVs may pass across the epithelial monolayer also paracellularly (Jones et al., 2020; Krsek et al., 2023) and come in contact with immune cells of the host, so we proceeded to investigate their immunoregulatory effects on innate immune cells. We analysed the interaction of MV6, MV12 and MV48 with the murine macrophage cell line RAW 264.7, as a representative of the host's first defence against undesirable antigens. We measured the production of RNS, as well as the release of pro‐inflammatory and immunoregulatory cytokines (TNFα, IL‐6 and IL‐10, respectively). To verify that the observed effects were caused by MVs, we used a series of dilutions of each MV pellet.

**FIGURE 3:**
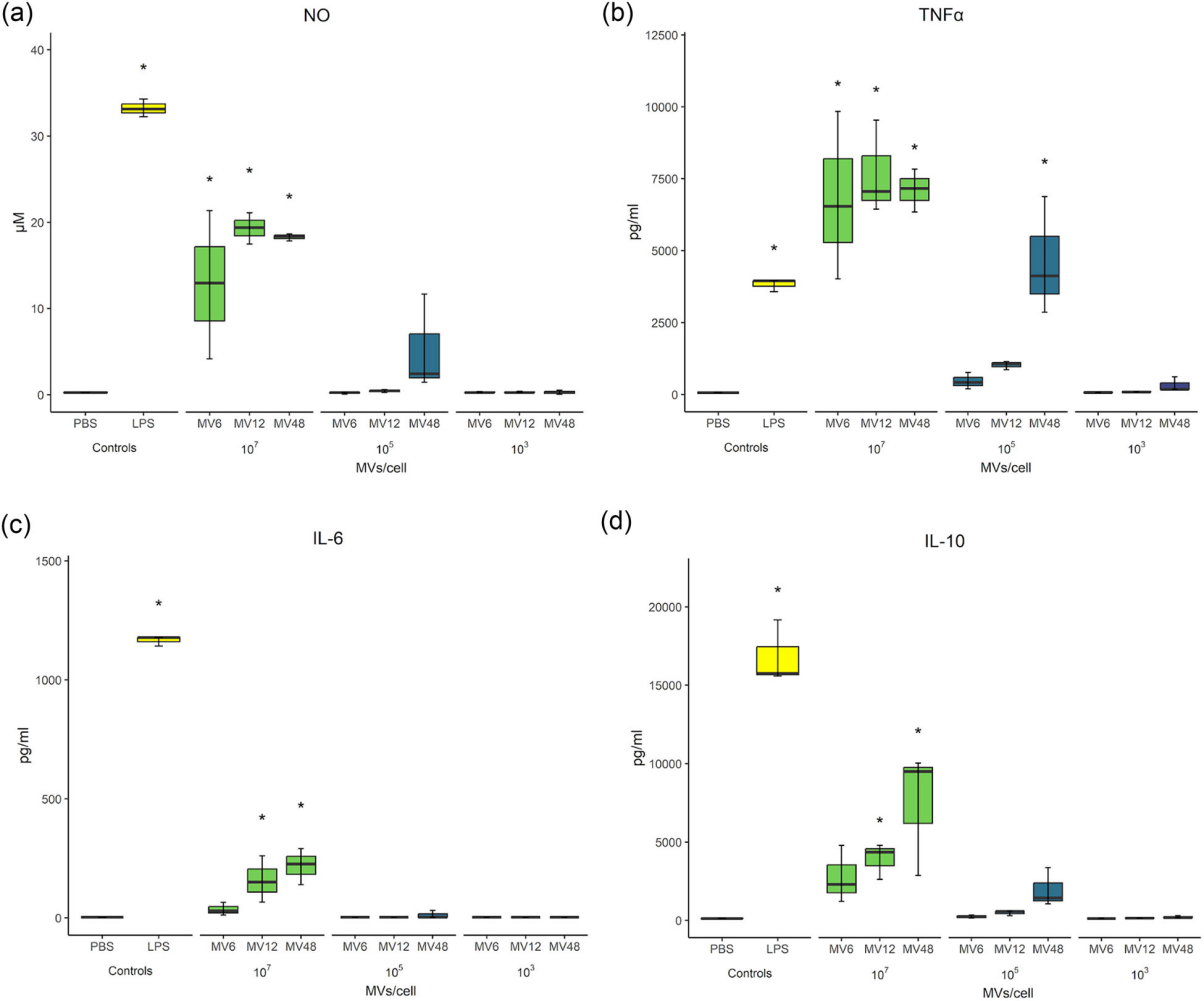
Pro‐inflammatory (NO, TNFα, IL‐6) and immunoregulatory (IL‐10) mediators (a–d, respectively) produced by RAW 264.7 treated with *Lacticaseibacillus rhamnosus* CCM7091 membrane vesicles (MVs) in three concentrations (10^7^, 10^5^ and 10^3^ MVs/cell). MVs were isolated at different times of cultivation: 6, 12 and 48 h (MV6, MV12, MV48); PBS was used as negative control (Ctrl), and Escherichia coli lipopolysaccharide (LPS) was used as positive control. The box plots show medians (lines), inter‐quartile ranges (boxes) and minimum and maximum values (whiskers); ANOVA was followed by Dunnett's multiple comparisons test versus negative control; statistical significance was assessed at * *p* < 0.05; *n* = 3.

Stimulation of RAW 264.7 cells by MV48 resulted in an increased inflammatory reaction, in a concentration‐dependent manner. The highest concentration of all the MVs (10^7^ MVs per cell), and MV48 in particular, induced the most significant increase in the analysed inflammatory mediators (RNS, TNFα and IL‐6) (Figure 3a–c). The middle concentration (10^5^ MVs per cell) revealed the differences between the MVs isolated from different stages of the *L. rhamnosus* growth curve compared with vehicle‐treated control – the production of pro‐inflammatory mediators was elevated after the MV48 treatment, in the case of TNFα statistically significantly. To get a more comprehensive view of the macrophages’ response to the MVs, we determined the production of the immunoregulatory cytokine IL‐10. Treatment with MV48 induced an enhanced production of IL‐10 (Figure 3d), corresponding with a heightened RNS and TNFα release at concentrations of 10^7^ and 10^5^ MVs per cell. MV6 caused a minor increase in the IL‐10 production which did not reach statistical significance, not even at the concentration of 10^7^ MVs per cell, compared to the vehicle‐treated control.

In summary, functional tests of *L. rhamnosus*‐derived MVs have shown that different‐time‐points MVs treatment of immune cells results in diverse responses. The MV48 elicits a pro‐inflammatory response of murine macrophages, as well as the production of immunoregulatory cytokine IL‐10, in contrast to MV6 and MV12.

### 3.4 The protein profile of *L. rhamnosus* CCM7091‐derived MVs changes with the growth phase of the maternal culture

**FIGURE 4:**
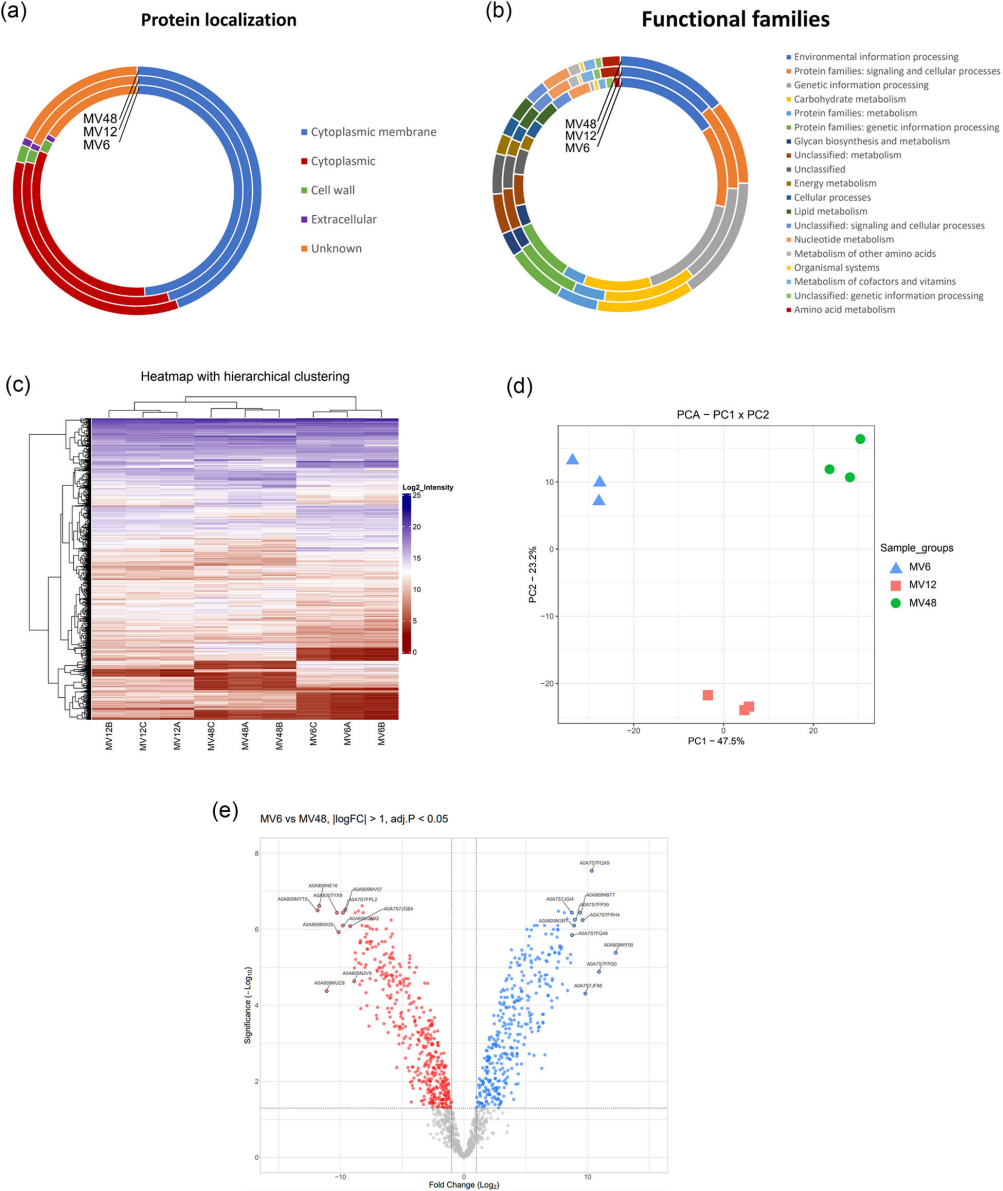
Proteomic analysis of *Lacticaseibacillus rhamnosus* CCM7091 membrane vesicles (MVs) isolated at different times: 6, 12 and 48 h (MV6, MV12, MV48). (a) Pie chart showing the relative proportions of colocalized proteins in all MV samples. Predictions were performed using PSORTb software. (b) Pie chart showing the distribution of individual functional families in all MV samples. Predictions were performed using KEGG Mapper. (c) Heatmap representation of clustering according to the protein abundance in MV samples. Normalized and imputed values were used. Heatmap was created using ComplexHeatmap package in R. Hierarchical clustering method was used. (d) Principal component analysis of MV samples created using ggplot2 package in R. (e) Volcano plot showing proteomics data from MV6 versus MV48. The dots indicate different proteins that display both large magnitude fold changes (*x*‐axis) and high statistical significance (−log10 of adjusted *p*‐values, *y*‐axis). The LIMMA package in R was utilized to calculate statistical significance. A log fold change >1 and an adjusted *p*‐value < 0.05 were chosen as the thresholds to determine differential expression (grey – unchanged, red – decreased, blue – increased).

To elucidate the cause of different immunomodulatory effects of the MVs isolated from different time points of the growth curve of *Lacticaseibacillus rhamnosus* CCM7091, we analysed their protein composition by LC‐MS/MS (each time point MV sample in triplicate). A total of 879, 1081 and 882 proteins were associated with MV6, MV12 and MV48, respectively. Each protein was identified based on at least two unique peptides and was present in at least one replicate. Protein localization was similar across the different time points samples (Figure 4a). The majority of the proteins were classified as either cytoplasmic membrane (MV6 = 429, MV12 = 491, MV48 = 393) or cytoplasmic (MV6 = 285, MV12 = 364, MV48 = 302) proteins (Table S3), based on the predictions made using the PSORTb software. In contrast, extracellular proteins and cell wall‐associated proteins were in the minority. Part of the identified proteins were annotated only as predicted or inferred from homology, hence the origin of about 18% (MV6 = 140, MV12 = 196, MV48 = 158) proteins was unknown.

Then, we determined the functional classification of MV6, MV12 and MV48 proteomes using the KEGG Mapper (Figure 4b). Again, since a significant proportion of proteins in our dataset had not yet been reviewed in databases such as UniProt and were annotated as either predicted or inferred from homology, only approximately 65% of the total proteins were classified into functional categories (MV6 = 65.9%, MV12 = 65.4%, MV48 = 66%) (Table S4). The overall number of functional KEGG categories of the *L. rhamnosus* CCM7091 MV‐associated proteins was 19. The majority of the proteins were assigned to Environmental information processing (MV6 = 10.6%, MV12 = 9.0%, MV48 = 9.4%), genetic information processing (MV6 = 11.1%, MV12 = 9.1%, MV48 = 10.4%), protein families: signalling and cellular processes (MV6 = 8.3%, MV12 = 7.9%, M48 = 7.1%) and carbohydrate metabolism (MV6 = 6.9%, MV12 = 8.4%, MV48 = 8.3%). The distribution of MV proteins between the three time point samples according to their functional characteristics was similar with only subtle differences. For instance, MV12 and MV48 had a slightly higher percentage of proteins belonging to carbohydrate metabolism and amino acid metabolism compared to MV6. On the other hand, MV6 contained a slightly higher proportion of genetic information processing proteins (Table S4).

The core proteome, common to all MV samples, consisted of 708 proteins (Figure S5). Surprisingly, the MV6 proteome comprises 43 unique proteins, mainly (61.3%) belonging to genetic information processing proteins. The MV12 proteome consisted of 110 unique proteins, of which 27.9% belong to genetic information processing proteins and 20.1% belong to carbohydrate metabolism. Lastly, MV48 contained 17 unique proteins of which only six were annotated.

We also quantitatively analysed the protein cargo and visualized it by heatmap clustering (Figure 4c). The biological triplicates clustered together, confirmed by PCA (Figure 4d) suggesting that protein loading into MV is a reproducible and selective process in *L. rhamnosus* CCM7091. Differences between samples based on protein intensities appeared to reflect the time‐point of the isolation. Significant changes in protein expression are depicted in volcano plots by fold change (|log_2_FC| > 1, adj. *p* < 0.05), where the biggest difference in protein intensities was observed between MV6 and MV48 (Figures 4e and S6). The 10 most significantly up‐ and down‐regulated proteins are annotated in each combination (Table S5). To sum up, the proteomic characterization of *L. rhamnosus‐*derived MVs showed significant differences between MV6 and MV48.

**FIGURE 5:**
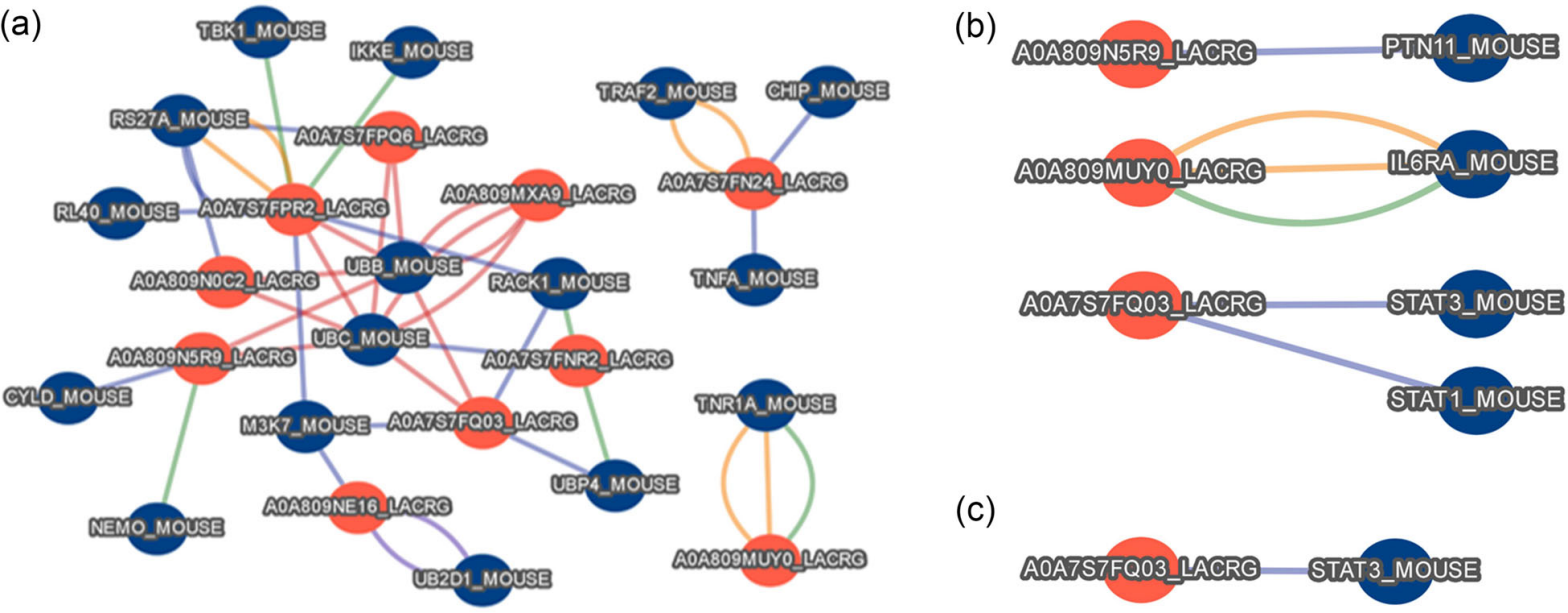
Predicted interaction network of up‐regulated proteins from *Lacticaseibacillus rhamnosus* CCM7091 membrane vesicles isolated after 48 h (MV48) with murine (a) TNFα signalling cascade proteins, (b) IL‐6 signalling cascade proteins and (c) IL‐10 signalling cascade proteins. Red dots represent *L. rhamnosus* CCM7091 proteins; Uniprot ID is used as a protein identifier. Blue dots represent host proteins; Uniprot name is used as a protein identifier. The interactions were predicted with host proteins, which were retrieved from the Reactome database (Milacic et al. 2024). The interolog prediction on the PredHPI Webserver (Loaiza & Kaundal 2021) was employed to predict interactions, with the criteria set to a minimum coverage of 60% and a sequence identity threshold of 30% for each interacting partner.

To investigate the cause of the effects we observed in vitro (the enhanced pro‐inflammatory mediators’ and IL‐10 production after MV48 treatment), we predicted the immunomodulatory properties of MV protein cargos in silico. We analysed PPI between MV proteomes and proteins involved in IL‐6, TNFα and IL‐10 signalling pathways. The MV48 proteome appears to have stronger immunomodulatory potential than MV6 and MV12, therefore we focused on the interaction of the MV48 up‐regulated proteins with the abovementioned pathways. Predicted PPI are summarized in Tables S6–S9. We identified interactions of the following proteins: Aminopeptidase (A0A809MUY0), Aminotransferase class V‐fold PLP‐dependent enzyme (A0A809MXA9), DUF72 domain‐containing protein (A0A809NAH1), Carboxypeptidase (A0A809NCU5), Threonyl‐tRNA synthetase (A0A7S7FPR2), Valyl‐tRNA synthetase (A0A809N5R9), Non‐specific serine/threonine protein kinase (A0A809N7A1), Seryl‐tRNA synthetase (A0A7S7FQ03), ABC transporter permease sub‐unit (A0A809NDK7), AAA domain‐containing proteins (A0A809N0C2, A0A7S7FPQ6), Thioredoxin reductase (A0A7S7FP57), Putative oxidoreductase (A0A809NE16), Glucose‐6‐phosphate 1‐dehydrogenase (A0A7S7FN24) and Malolactic protein (A0A7S7FNR2) (Figures 5 and S7 and Tables S6–S9). The expression of these proteins is at least twice increased in MV48 proteome compared to MV6 and MV12, showing the possible contribution to the functional differences between MV isolated at different times.

### 3.5 The LTA participates in the immunomodulatory properties of the *L. rhamnosus* MVs

**FIGURE 6:**
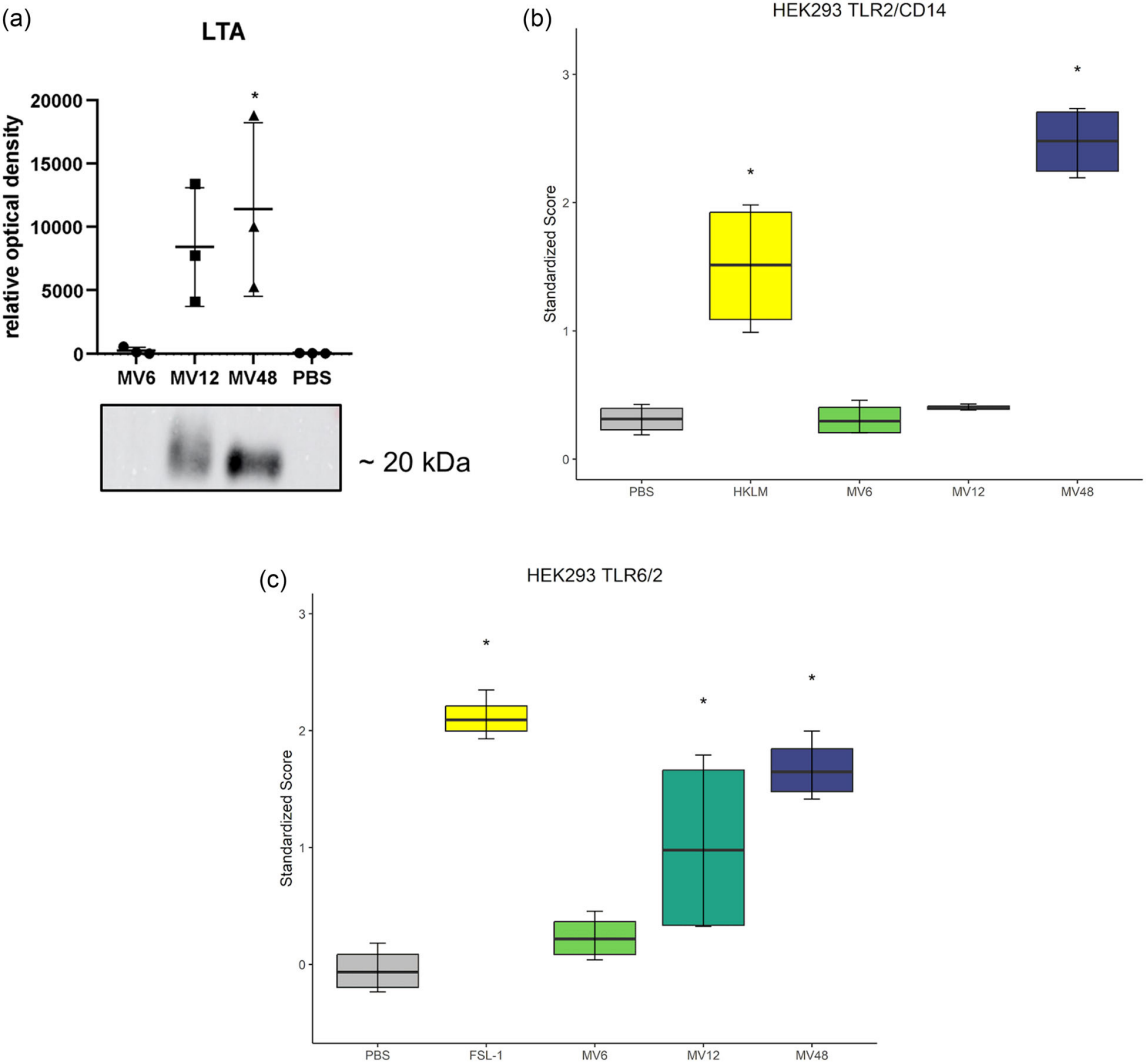
(a) Relative optical density and representative Western blot of lipoteichoic acid (LTA) detected in *Lacticaseibacillus rhamnosus* CCM7091 membrane vesicles (MVs) isolated after 6, 12, 48 h (MV6, MV12, MV48). PBS was used as a negative control. Bars represent means ± SD; statistical significance was assessed at * *p* < 0.05; ordinary one‐way ANOVA was followed by Dunnett's multiple comparison test versus negative control; *n* = 3. (b), (c) Human cell receptors involved in the recognition of *Lacticaseibacillus rhamnosus* CCM7091 membrane vesicles (MVs). Effects of MVs isolated after 6, 12, 48 h (MV6, MV12, MV48) on activation of TLR2/CD14 (a) and TLR6/2 (b) expressed on human embryonic kidney cells HEK‐293 (concentration 10^5^ MVs/cell). The recognition abilities are compared to the negative control (PBS), and appropriate positive controls are depicted (HKLM for HEK293 TLR2/CD14, FSL‐1 for HEK293 TLR6/2). Results were evaluated based on IL‐8 production activity in cell media. The box plots show medians (lines), inter‐quartile ranges (boxes) and minimum and maximum values (whiskers); data normalization was performed using *Z*‐score; statistical analysis was performed using the ANOVA and the significance was assessed at *p < 0.05; *n* = 4.

To expand the search for the possible cause of different immunomodulatory properties of various‐time‐points MVs beyond the protein composition, we detected the expression of the Gram‐positive bacteria marker, LTA, by Western blot and immunodetection. The overall LTA levels in the MVs seemed to increase with the longer periods of maternal bacteria cultivation (Figure 6a). However, only the MV48 exerted a significantly higher expression of LTA compared to the negative control.

### 3.6 TLR2 is involved in the recognition of the late‐stationary‐phase *L. rhamnosus* MVs

Having shown that the MV48 contain the LTA, we went further and attempted to identify the molecular mechanism of the MVs’ interaction with the target cells. Generally, bacterial components are recognized by the pattern recognition receptors (PRRs) of the host cells. To reveal the specific receptors involved in MV recognition in target cells, we analysed the interaction of MV6, MV12 and MV48 with HEK‐293 cells over‐expressing relevant TLRs and NOD‐like receptors.

TLR2 is known to heterodimerize with other TLRs (TLR1/2 and TLR2/6), a property believed to extend the range of PAMPs that TLR2 can recognize, including LTA, peptidoglycan and diacylated mycoplasmal lipopeptide, triacylated lipopeptides and others. Furthermore, pathogen recognition by TLR2 is strongly enhanced by CD14.

Stimulation of HEK‐293 cells transfected with TLR2/CD14 and TLR6/2 by MV48 resulted in significant enhancement of IL‐8 production compared with vehicle‐treated control (Figure 6b,c). None of the MVs isolated from different stages of the *L. rhamnosus* CCM7091 growth curve activated TLR1/2‐triggered signalling pathway and scrambled control (293/null cells) (Figure S8a,b).

Additionally, we tested the activation of NOD1 and NOD2 – receptors participating in the recognition of molecular patterns that are derived from peptidoglycan, one of the components of cell walls of Gram‐positive bacteria (Kurata et al., 2022). Our results did not confirm the significant stimulation of NOD1 and NOD2 receptors by MV6, MV12 or MV48 (Figure S8c,d).

Further, the Gram‐positive bacteria and their vesicles should not contain LPS in their structure. We confirmed this by the absence of TLR4A‐MD2‐CD14 cell response after treatment with the *L. rhamnosus* CCM7091‐derived MVs (Figure S8e). Summarized, TLR2 and TLR2/6 appear to play an exclusive role in the activation of immune response by MVs in the later stages of the *L. rhamnosus* CCM7091 growth curve.

## 4 DISCUSSION

The therapeutic potential of bacterial MVs remains to be fully exploited – the most discussed possibilities include postbiotics, drug delivery carriers and vaccine platforms. Compared with OMVs, the MVs produced by *Lacticaseibacillus* spp. and other GRAS Gram‐positive bacteria emerge as even more promising, due to the absence of endotoxins (therefore no need to artificially remove them (Kim et al., 2017; Park et al., 2021)) while keeping the general imunoboosting properties. Yet, the studied MVs are being harvested at different stages of bacterial growth (Choi et al., 2022; Han et al., 2023; Keyhani et al., 2022; Kurata et al., 2022) and very little is known about the influence of the bacterial growth phase on the MVs properties. To the best of our knowledge, only three studies on the cultivation time dependence of Gram‐positive bacteria‐derived MVs have been published to date (da Luz et al., 2022; Lee et al., 2021; Mehanny et al., 2022). The authors compare the effects of two (da Luz et al., 2022; Mehanny et al., 2022) or three (Lee et al., 2021) time points of the growth curve, focusing on either protein content (da Luz et al., 2022), immunomodulatory effects (Mehanny et al., 2022) or antibacterial activity (Lee et al., 2021). However, the effects of the overall growth stages on the composition, uptake by recipient cells, signalling via PRRs and immunomodulatory properties of the MVs, considering the actual growth curve of the studied bacterial species, are lacking.

In our work, we aimed to cover the entire growth curve of *L. rhamnosus* CCM7091: the early exponential, the late exponential and the late stationary phase. We looked at the MVs in a comprehensive manner, describing their biophysical properties, uptake by epithelial cells, proteomic composition, immunomodulatory effects and recognition by PRRs, to evaluate their potential for application as drug carriers.

From a biophysical point of view, MV6 seems to differ the most from the rest of the groups – both the yield and the average diameter were the lowest, whereas the MV48 diameter was significantly bigger compared to the MV6. These findings are in accordance with Mehanny et al. (2022), who showed higher yields and a higher size range of MVs isolated at a later stage of *Streptococcus pneumoniae* growth, although comparing stationary‐phase with death‐phase MVs (Mehanny et al., 2022). Similarly, Lee et al. (2021) observed an increased particle concentration with increasing cultivation time, although they did not detect any change in the particle diameter of *L. plantarum‐*derived MVs (Lee et al., 2021). Their results suggest that the yield of MVs improves with longer cultivation, as the MVs probably cumulate in the medium throughout the longer cultivation. Interestingly, we have shown that the highest increase in MV yield occurs in the late‐exponential and not necessarily in the late‐stationary‐phase MVs. This information may be determinative for the maximization of MV yields for future medical applications. We assume that the decrease in the presence of MVs in the late stationary phase of the growth curve may be explained by their uptake by the maternal bacteria (Kim et al., 2016). Another reason may be their aggregation or fusion, hence the lower numbers and higher average particle diameter. These hypotheses deserve additional experiments to be validated.

To our best knowledge, here we provide for the first time proof that MV uptake efficiency is dependent on the length of the maternal cell cultivation. To reflect the effect of MVs in the context of the intestinal tract, the typical habitat of *L. rhamnosus*, we investigated the route of their uptake by intestinal epithelial cells. We confirmed the previous findings (Champagne‐Jorgensen et al., 2021) that the *Lacticaseibacillus* spp. MVs are taken up by intestinal cells likely by clathrin‐dependent endocytosis. On top of that, as we employed a wider variety of inhibitors, we showed that the MV uptake routes are not limited to the clathrin‐mediated endocytosis, but also the lipid rafts‐mediated internalization and micropinocytosis take place. Contrarily to Bajic et al. (2020), in our hands, the predominant uptake route was the caveolae‐mediated endocytosis. This may be explained by species specificity (*L. rhamnosus* vs. *L. plantarum*), and target cells diversity (Caco‐2 vs. HT29).

It was proven that both Gram‐positive and Gram‐negative bacteria‐derived EVs can modulate the host immune response (Chiba et al., 2010; Kurata et al., 2022; Mehanny et al., 2022); the OMVs containing LPS on the surface may be an even stronger modulator of the innate immune response than the source bacteria (Gilmore et al., 2021). Therefore, we investigated the composition and immunomodulatory effects of the *L. rhamnosus*‐derived MVs In vitro, as the starting point for future medical tests. We found out that the effects of MVs are dose‐dependent, with 10^5^ MVs/cell being the concentration revealing the biggest differences between different time points MVs in macrophages. In agreement with Mehanny et al. (2022), at this concentration, we observed a significant increase in the production of TNFα by macrophages when treated with late‐stage MVs (Mehanny et al., 2022). Similarly, the early‐stages MVs induced TNFα and IL‐6 production, in our hands, however, the effect was statistically significant only at the concentration of 10^7^ MVs per cell. It is noteworthy that the highest MV concentration resulted in an overall increase in TNFα and RNS in all time‐point MVs. This highlights the importance of standardized MV dosing for in vitro experiments and the need for concentration titration. We also measured the release of the immunoregulatory cytokine IL‐10, which showed an increase after MV48 treatment, as the cells probably regulate the inflammatory response in a negative feedback loop (Morhardt et al., 2019).

To examine the cause of the different immunomodulatory effects by the specific time‐point MVs, we analysed their proteome and the LTA content. The proteomes of *L. rhamnosus*‐derived MVs consisted of around 1000 identified proteins, mainly belonging to the cytoplasmic membrane and cytoplasmic proteins. The protein localization is in agreement with (da Luz et al., 2022; Hu et al., 2021; Kim et al., 2016; Tartaglia et al., 2020), whereas the number of identified proteins varies in these studies, probably caused by less sensitive proteomic profiling, more stringent bioinformatic data processing or maternal bacterial strain.

The distribution of MV proteins according to their functional characteristics was similar in all samples with subtle differences. Notably, the MV12 and MV48 proteomes contain a slightly higher percentage of proteins belonging to carbohydrate and amino acid metabolisms compared to the MV6 proteome. This may be attributed to the continued metabolic activity of cells during the transition from exponential to the stationary phase when *L. rhamnosus* cells gain energy from free amino acids or utilize different carbohydrates under environmental stress (De Angelis et al., 2016; Laakso et al., 2011). During the stationary phase of *L. rhamnosus* GG, alternative enzymes related to the tagatose‐6‐phosphate and Leloir pathways are produced, leading to the hydrolysis of alternative carbohydrates. This is connected with a decrease in glucose concentration at the end of the exponential growth phase (Chubukov & Sauer, 2014). Consistent with this, we observed increased protein intensities in the carbohydrate metabolisms functional group (e.g. tagatose‐6‐phosphate kinase, tagatose 1,6‐diphosphate aldolase, galactose mutarotase, galactokinase) in MV12, reflecting physiological changes of maternal cells at the end of the exponential phase and persisting in MV48. Interestingly, the MV6 proteome contains 43 unique proteins, most of them related to the genetic information processing functional group, in alignment with their reported increased expression during the exponential growth phase and subsequent decrease in the stationary phase (Veselovsky et al., 2022). This confirms that MVs mirror the physiological state of the maternal culture. Besides, the abundance of proteins related to the transfer of various molecules and metabolic processes confirmed the carrier function of the MVs.

In silico PPI analyses revealed potential interactions between certain bacterial proteins and the host proteome. We focused on proteins up‐regulated in MV48, as the MV48 elicited the strongest immune reaction. These bacterial proteins include Aminopeptidase (A0A809MUY0) (predicted to interact with IL‐6 receptor subunit α and TNF receptor superfamily) and Aminotransferase class V‐fold PLP‐dependent enzyme (A0A809MXA9) (predicted to interact with a protein associated with endocytosis and vesicle scission (Q05193)). Additionally, we identified DUF72 domain‐containing protein (A0A809NAH1) and Carboxypeptidase (A0A809NCU5), which are predicted to interact with NFκ‐B subunit, related to multiple cell processes, including inflammation and immune response. Besides, several Aminoacyl‐tRNA synthetases (ARSs), such as Threonyl‐RS (A0A7S7FPR2), Valyl‐RS (A0A809N5R9) and Seryl‐RS (A0A7S7FQ03), but also non‐specific serine/threonine protein kinase (A0A809N7A1), ABC transporter permease subunit (A0A809NDK7), AAA domain‐containing proteins (A0A809N0C2, A0A7S7FPQ6), Thioredoxin reductase (A0A7S7FP57), Putative oxidoreductase (A0A809NE16), Glucose‐6‐phosphate 1‐dehydrogenase (A0A7S7FN24) and Malolactic protein (A0A7S7FNR2) were identified. ARSs are known for their role in protein synthesis, linking amino acids to transfer RNAs. They contribute to bacterial metabolism and virulence (Baddal et al., 2015; Kirubakar et al., 2018; Maloney et al., 2009) and certain bacterial ARSs have been implicated in various immune responses (Nie et al., 2019). *Leishmania donovani* TyrRS could be released from the parasite cytoplasm to the outside of the cell and specifically bind to the host macrophages thereby promoting the secretion of TNF‐ɑ and IL‐6 (Bhatt et al., 2011). *Brugia malayi* asparaginyl‐RS has also been shown to be closely related to the host immune response (Hameed et al., 2019; Ramirez et al., 2006). This corresponds with MV48 effects on murine macrophages we observed in vitro, suggesting that ARSs contribute to the MVs’ immunomodulatory effects. Briefly, our results and PPI analyses suggest that the changes in the proteomic cargo of the MVs isolated at different growth stages may contribute to their different immunomodulatory capacities by interacting with TNFα, IL‐6 and IL‐10 signalling pathways.

MVs were isolated from a batch cultivation system, implying that MV48 should encompass information from MV6 and MV12 if present in the medium. Surprisingly, unique proteins found in MV6 and MV12 were not identified in MV48, which raises questions for further investigation. Hypotheses for future research include continuous MV uptake by cells (Kim et al., 2016) serving for a constant exchange of information, potentially affected by nutrient scarcity, leading to decreased MV48 proteomic variety. Alternatively, unique MV surface proteins might undergo increased degradation in the presence of proteolytic enzymes during bacterial growth, especially under stress conditions, possibly resulting in the release of lytic enzymes (Frees et al., 2007) and also because the MVs’ biogenesis mechanism of explosive cell lysis (proven in Gram‐positive bacteria by Jeong et al. (2022)) may become more eminent when the bacterial culture is under stress conditions in late‐stationary phase (Turnbull et al., 2016). This theory aligns with the observed reduced bacterial cell numbers.

To further explore the diverse immunomodulatory effects observed in vitro, we were interested in whether the LTA, a Gram‐positive bacteria marker, may contribute to the effects of MVs. In *Staphylococcus aureus*, it was confirmed that LTA is more abundant in MVs compared to the secretome (Uppu et al., 2023). The LTA is also known to be built differently in the cell membrane during different growth stages (Shiraishi et al., 2018), however, to the best of our knowledge, no such information was confirmed in the LTA content in MVs isolated at different time points of bacteria cultivation. Here, we have shown for the first time that the LTA amounts in the MVs indeed differ and increase with longer cultivation time. We suppose that the LTA contributes significantly to the immunoregulatory effects in the murine macrophages, being most abundant and supposedly potent in the *L. rhamnosus*‐derived MVs isolated in the late stationary phase of the growth curve.

Based on the LTA contents, we continued the search for the mechanism of action of the MVs by using HEK293 cells overexpressing relevant PRRs. In agreement with Champagne‐Jorgensen et al. (2021) and Kurata et al. (2022), we confirmed that not only *L. plantarum*, but also *L. rhamnosus* MVs activate cell response by TLR2. In their work, Kurata et al. (2022) did not detect the LTA in *L. plantarum* and identified different ligands recognized by TLR2, still, it is generally known that the LTA is recognized by TLR2/6 which we confirmed here. The study of Friedrich et al. showed that the LTA isolated from *L. rhamnosus* GG induced dendritic and T cells activation, supposedly by TLR2 signalling pathway (Friedrich et al., 2022). This corresponds with our results that the increased amount of LTA in MV48 correlated with the increased TLR2/CD14 and TLR6/2 signalling, suggesting that the LTA is a key immunomodulatory molecule abundant particularly in the late stages‐of‐the‐growth‐curve MVs as we have found out in this research. Contrastingly to Kurata et al. (2022), however, in our hands, none of the MVs induced significant NOD1 nor NOD2 activation. These receptors recognize peptidoglycan‐derived molecular patterns, so a possible cause of the discrepancy is that peptidoglycan may be only a co‐isolate, which we removed in the MV purification step. To conclude, the late‐stationary‐phase *L. rhamnosus* MVs have suitable immunoboosting properties for further therapeutical applications.

Overall, here we addressed the pressing need for characterization and standardization of the MVs, to be fully utilized in clinics. We have described the profile of *L. rhamnosus*‐derived MVs and how they change throughout bacterial growth. A limitation of our study is related to the incomplete annotation of the proteomic databases in the bacterial proteins we used for proteomic analysis (around 65% of the identified proteins were properly annotated). Therefore, the MV‐protein localization and their classification into functional groups may be influenced by the unknown proteins. Also, the nucleic acids and lipids contained in the MVs, which were not at the centre of the interest of our study, may contribute to the effects of MVs on the host cells and we plan to focus on them in our future research. Lastly, in the late stationary phase, a few bacterial cells may start to die, causing a possible small portion of membrane components of the dying cells that may be co‐isolated with the MVs (Liu et al., 2022) or bacterial ghosts to be present in the MV48 sample. Bacterial ghosts are empty cell envelopes and have been recently proposed, together with the MVs, as a promising therapeutic delivery system (Alexander & van Pijkeren, 2023; Yu et al., 2022). Incapable of replication, their presence cannot be revealed in a sterility test, so we cannot rule out their presence in the MV samples.

## 5 CONCLUSION

In this study, we demonstrated that the growth phase of the maternal bacterial culture impacts the size, composition, biophysical and immunomodulatory properties of *L. rhamnosus*‐derived MVs. Therefore, the MV harvest point should be considered and standardized in future research, especially when aiming at reproducibility and therapeutic use of the MVs. Our cultivation and MV isolation procedure is highly reproducible, illustrated by the similitudes of the triplicates, both in MADLS and proteomics results. We further reported that late‐stationary‐phase *L. rhamnosus* MVs are bigger, taken up predominantly by caveolae‐mediated pathway, and stimulate the immune response of murine macrophages. Our proteomic and PPI analyses indicate that the MV protein cargo changes with different isolation time points, probably contributing to their diverse immunomodulatory potentials. Here we showed for the first time that the expression of the LTA in the *L. rhamnosus* MVs increases with a longer cultivation period. The late‐stationary MVs further elicit the TLR2‐triggered signalling pathway, suggesting the contribution of LTA to the immunomodulatory effects of the MVs. Additionally, the MVs, compared with the maternal bacteria, exhibit much higher safety, and inability to replicate while still being effective immunoboosters. Therefore, for future medical applications of the probiotic bacteria‐derived MVs, the late‐stationary‐phase MVs may be the most suitable for effective immunoboosting while keeping all their safety properties. Further studies are needed to validate our findings on another probiotic Gram‐positive bacterial species and to eventually elucidate the contribution of nucleic acids to the MV's immunostimulatory effects.

## AUTHOR CONTRIBUTIONS

**Miriam Sandanusova**: Conceptualization; writing—original draft; writing—review and editing; investigation; data curation; formal analysis. **Kristyna Turkova**: Conceptualization; writing—original draft; writing—review and editing; investigation; project administration. **Eva Pechackova**: Investigation; data curation; methodology. **Jan Kotoucek**: Investigation; data curation; methodology. **Pavel Roudnicky**: Data curation; methodology. **Martin Sindelar**: Methodology; software. **Lukas Kubala**: Supervision; writing—review and editing. **Gabriela Ambrozova**: Conceptualisation; project administration; supervision; methodology; writing—review and editing.

## CONFLICT OF INTEREST STATEMENT

The authors declare no conflicts of interest.

## Supporting information

Supporting Information

## Data Availability

The data supporting the findings in this study are included in the article and are available from the authors upon reasonable request.
