# Supplementary material for: Growth phase matters: Boosting immunity via Lacticasebacillus‐derived membrane vesicles and their interactions with TLR2 pathways
Source: J Extracell Biol. 2024 Aug 22;3(8):e169. doi: 10.1002/jex2.169 (PMC11341917; doi:10.1002/jex2.169)
Supplement: Supplementary file 1 — Supporting Information [file JEX2-3-e169-s001.docx]

**Supplementary material**

**Growth phase matters: Boosting immunity via *Lacticaseibacillus*-derived membrane vesicles and their interactions with TLR2 pathways**

Miriam Sandanusova^1,2^, Kristyna Turkova^2^, Eva Pechackova^3^, Jan Kotoucek^4^, Pavel Roudnicky^5^, Martin Sindelar^1^, Lukas Kubala^1,2^, Gabriela Ambrozova^2^

*^1^Faculty of Science, Department of Experimental Biology, Masaryk University, Brno Czech Republic*

*^2^Department of Biophysics of Immune System, Institute of Biophysics of the Czech Academy of Sciences, Brno, Czech Republic*

*^3^Faculty of Science, Department of Biochemistry, Masaryk University, Brno, Czech Republic*

*^4^Department of Pharmacology and Toxicology, Veterinary Research Institute, Brno, Czech Republic*

*^5^Central European Institute of Technology (CEITEC), Masaryk University, Brno, Czech Republic*

Corresponding author: Gabriela Ambrozova, email: [ambrozova@ibp.cz](mailto:ambrozova@ibp.cz), Institute of Biophysics of the Czech Academy of Sciences, Kralovopolska 135, 61200 Brno, Czech Republic

Miriam Sandanusova and Kristyna Turkova contributed equally to this study and should be considered joint first authors.

**Materials and Methods**

**Proteomic sample preparation and analysis by liquid chromatography–tandem mass spectrometry**

Membrane vesicles of *Lacticasebacillus rhamnosus* CCM7091 were lysed in SDT buffer (4% SDS, 0.1M DTT, 0.1M Tris/HCl, pH 7.6) in a thermomixer (Eppendorf ThermoMixer® C, 30 min, 95°C, 750 rpm). After that, samples were centrifuged (15 min, 20 000 x *g*) and the supernatants (ca 50 μg of total protein) used for filter-aided sample preparation (FASP) as described elsewhere [1] using 0.5 μg of trypsin (sequencing grade; Promega). Resulting peptides were analysed by LC-MS/MS.

LC-MS/MS analyses of all peptides were done using UltiMate 3000 RSLCnano system (Thermo Fisher Scientific) connected to timsTOF Pro spectrometer (Bruker). Prior to LC separation, tryptic digests were online concentrated and desalted using trapping column (Acclaim™ PepMap™ 100 C18, dimensions 300 μm ID, 5 mm long, 5 μm particles, Thermo Fisher Scientific). After washing of the trapping column with 0.1% formic acid (FA), the peptides were eluted (flow rate – 300 nl/min) from the trapping column onto an analytical column (Aurora C18, 75μm ID, 250 mm long, 1.6 μm particles, heated to 50°C, Ion Opticks) by 60 min linear gradient program (3-80% of mobile phase B; mobile phase A: 0.1% FA in water; mobile phase B: 0.1% FA in 80% ACN). Equilibration of the trapping column and the analytical column was done prior to sample injection to sample loop. The analytical column was placed inside the Butterfly Heater (Phoenix S&T) and its emitter side was installed inside the CaptiveSpray ion source (Bruker) according to the manufacturer instructions with the column temperature set to 50 °C.

MSn data were acquired in data independent acquisition (DIA) mode with base method m/z range of 100-1 700 and 1/k0 range of 0.6-1.6 V×s×cm^-2^. Precursor range was defined to m/z 400-1 000 with equal windows sizes of 26 Th using two steps each PASEF scan and cycle time of 100ms locked to 100% duty cycle.

DiaPASEF data were processed in DIA-NN (version 1.8)[2] in library free mode against modified cRAP database (based on <http://www.thegpm.org/crap>; 111 sequences in total) and UniProtKB protein database for *Lactobacillus rhamnosus* ATCC 53103 (<https://www.uniprot.org/proteomes/UP000002067>; version 2022/12, number of protein sequences: 2,718). No variable, carbamidomethylation as fixed modification and trypsin/P enzyme with 1 allowed missed cleavages were set during the library preparation. False discovery rate (FDR) control was set to 1% FDR. MS1 and MS2 accuracies as well as scan window parameters were set based on the initial test searches (median value from all samples ascertained parameter values). MBR was switched on.

Protein MaxLFQ intensities reported in the DIA-NN main report file were further processed using the software container environment (<https://github.com/OmicsWorkflows>), version 4.6.3a. Processing workflow is available upon request. Briefly, it covered: a) removal of low-quality precursors and contaminant protein groups, b) protein group intensities log2 transformation and normalization (loessF), c) filtering out of protein groups not quantified in at least 2 replicates of single sample type, d) imputation of missing values using R package imp4p, e) differential expression analysis using LIMMA statistical test.


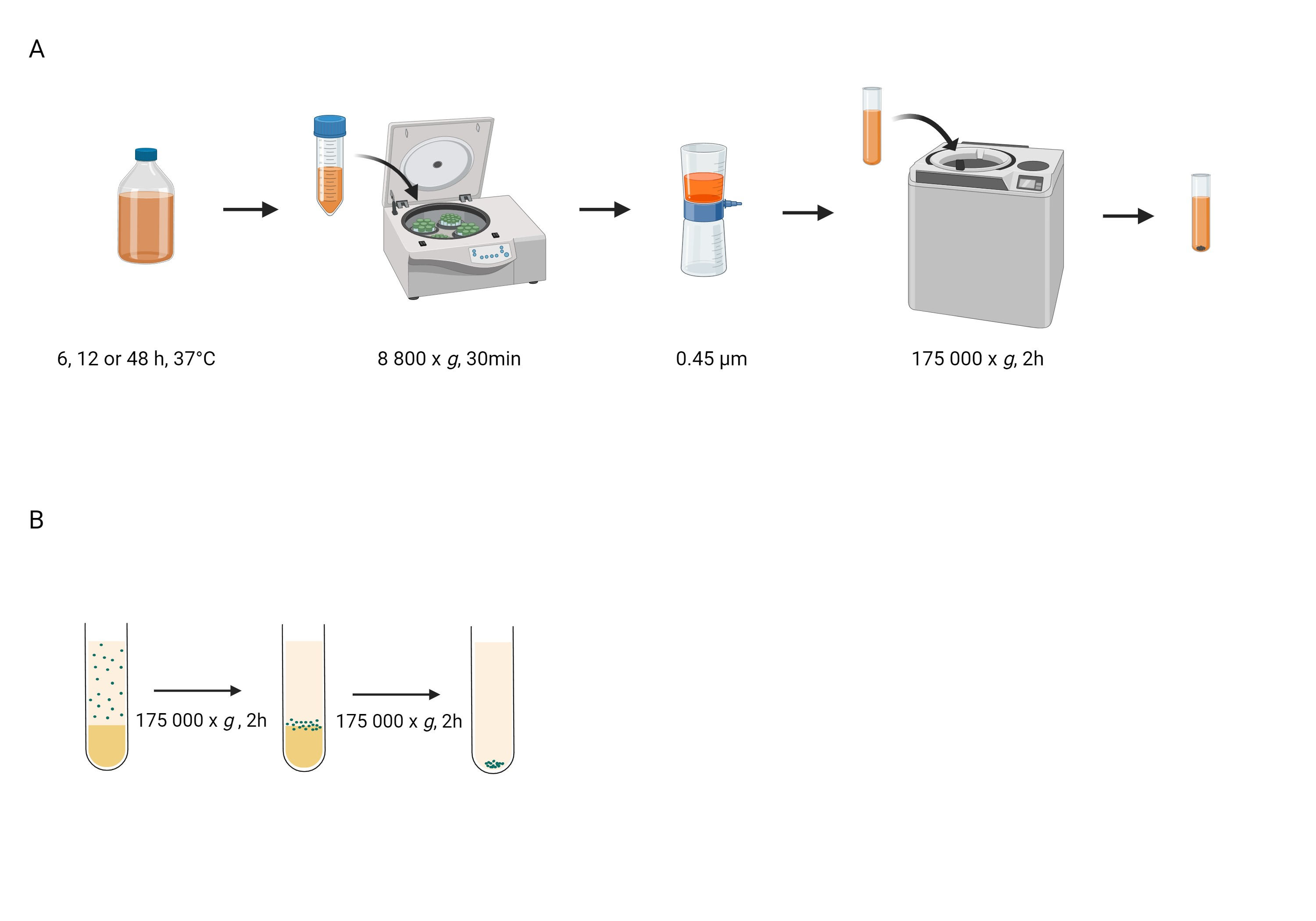


***Figure S1: Isolation (A) and purification (B) of Lacticaseibacillus rhamnosus-derived membrane vesicles.***

***Figure S2: Growth curve of Lacticaseibacillus rhamnosus CCM7091 cultivated in MRS broth at 37 °C for 48 h aerobically****. Data are shown as mean ± SD; n=3.*

***Figure S3: Size distribution by Intensity of Lacticaseibacillus rhamnosus CCM7091 membrane vesicles (MVs) isolated throughout bacterial growth (MV6, MV12, MV48).***

*
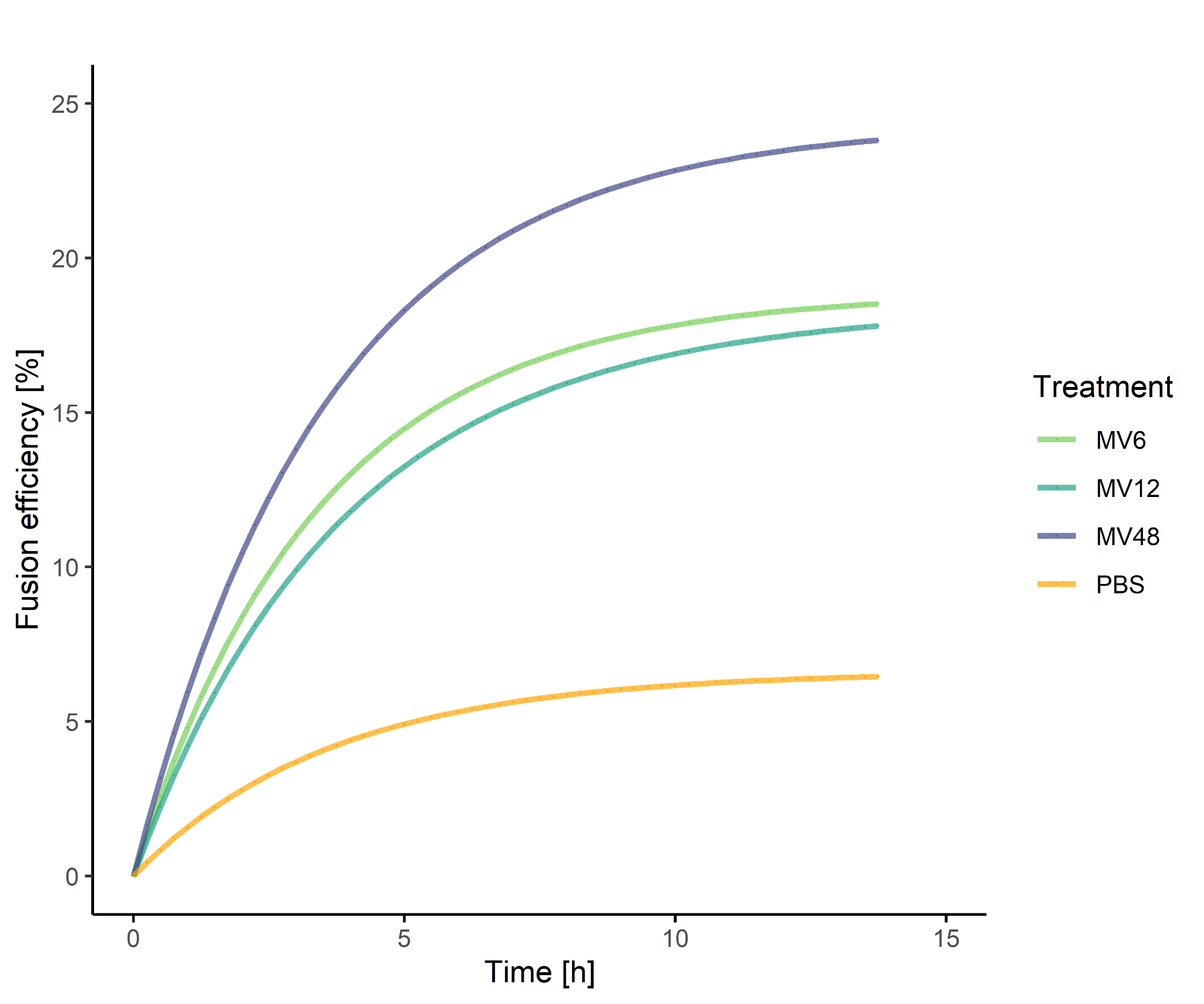
*

***Figure S4: Time-course dynamics of internalization of MVs into Caco-2 cells.*** *The efficiency of fusion events within Caco-2 cells (calculated according to Equation 1). The increase of the fluorescence signal was observed after addition of R-18 labeled MVs from different times of isolation (MV6, MV12, MV48) to Caco-2 cells in time. The vehicle alone, subjected to the same labeling protocol, was used as negative control (PBS).*

*
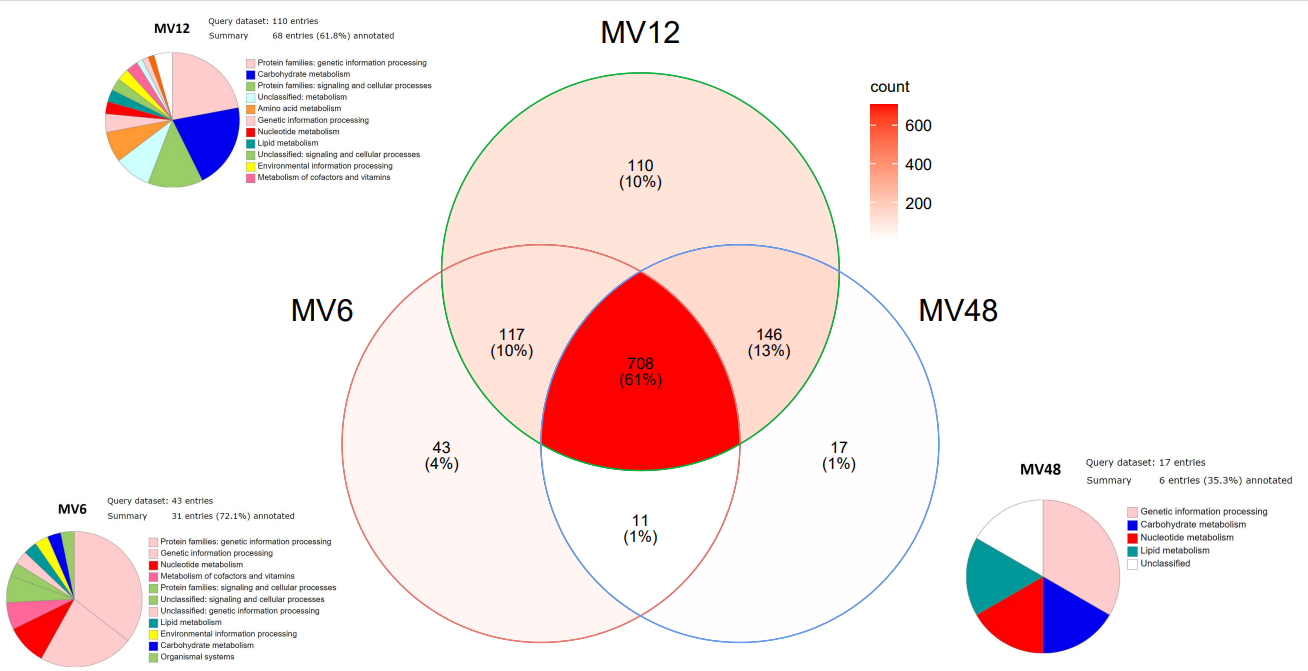
*

***Figure S5: Venn diagram depicting protein overlap of the membrane vesicles (MVs) samples.*** *The diagram was generated using the ggVennDiagram package in R. Functional protein categories are visualized using pie charts generated by KEGG Mapper. Number of entries reflects the information available in databases.*

A


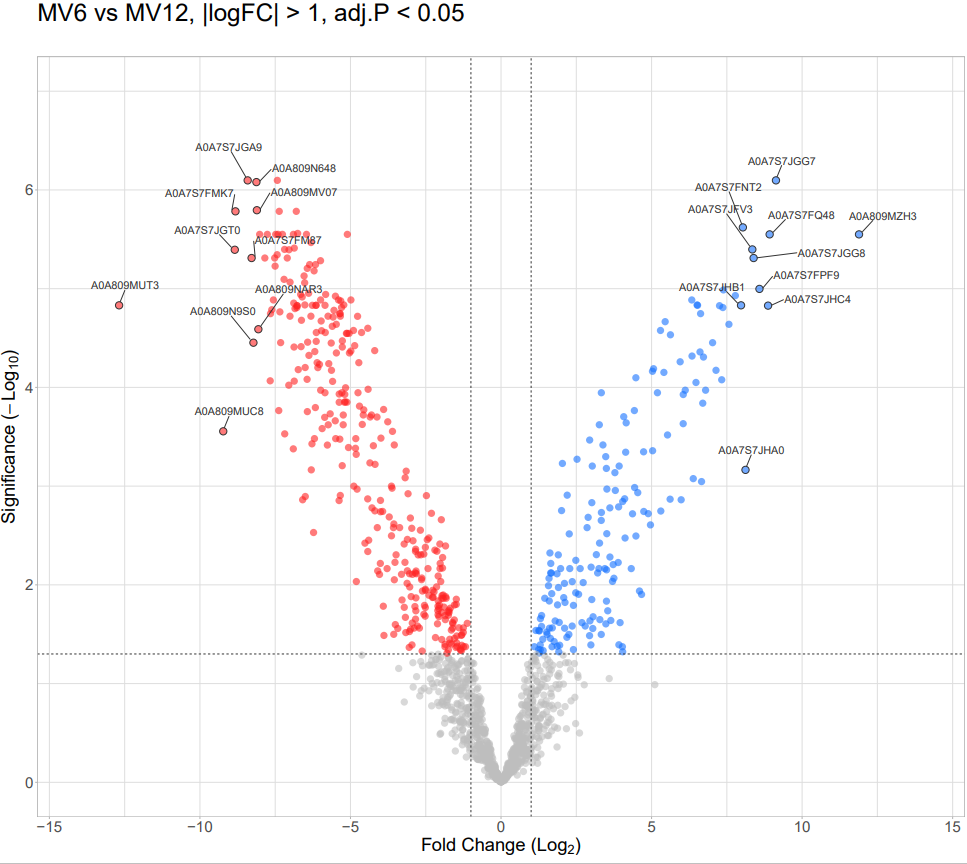


B


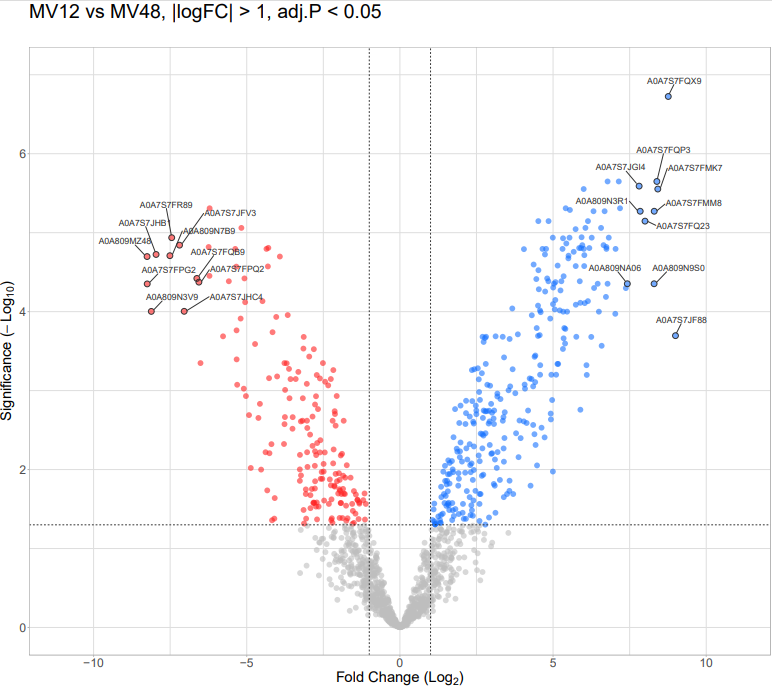


***Figure S6: Volcano plots showing proteomics data from membrane vesicles (MVs): MV6 vs MV12 (A) and MV12 vs MV48 (B).*** *The dots indicate different proteins that display both large magnitude fold changes (x axis) and high statistical significance (-log10 of adjusted p values, y axis). The LIMMA package in R was utilized to calculate statistical significance. A log fold change greater than 1 and an adjusted P value less than 0.05 were chosen as the thresholds to determine differential expression (grey – unchanged, red – decreased, blue – increased).*


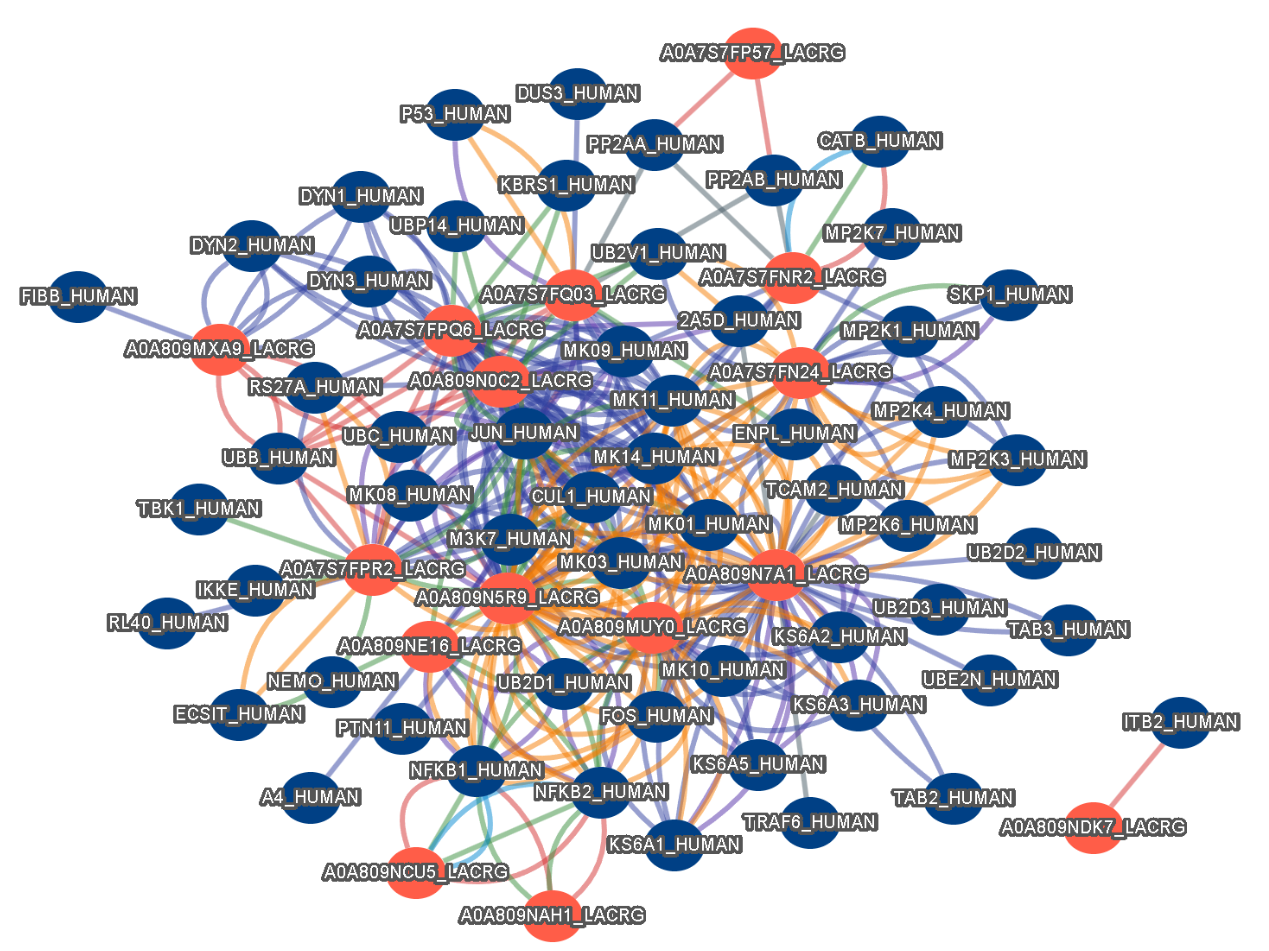


***Figure S7: Predicted interaction network of upregulated proteins from MV48 with human TLR cascade.*** *Red dots represent L.rhamnosus CCM7091 proteins; Uniprot ID is used as protein identifier. Blue dots represent host proteins; Uniprot name is used as a protein identifier. The interactions were predicted with proteins from the human TLR cascade, which were retrieved from the Reactome database [3]. The interolog prediction on the PredHPI Webserver [4] was employed to predict interactions, with the criteria set to a minimum coverage of 60% and a sequence identity threshold of 30% for each interacting partner.*

A
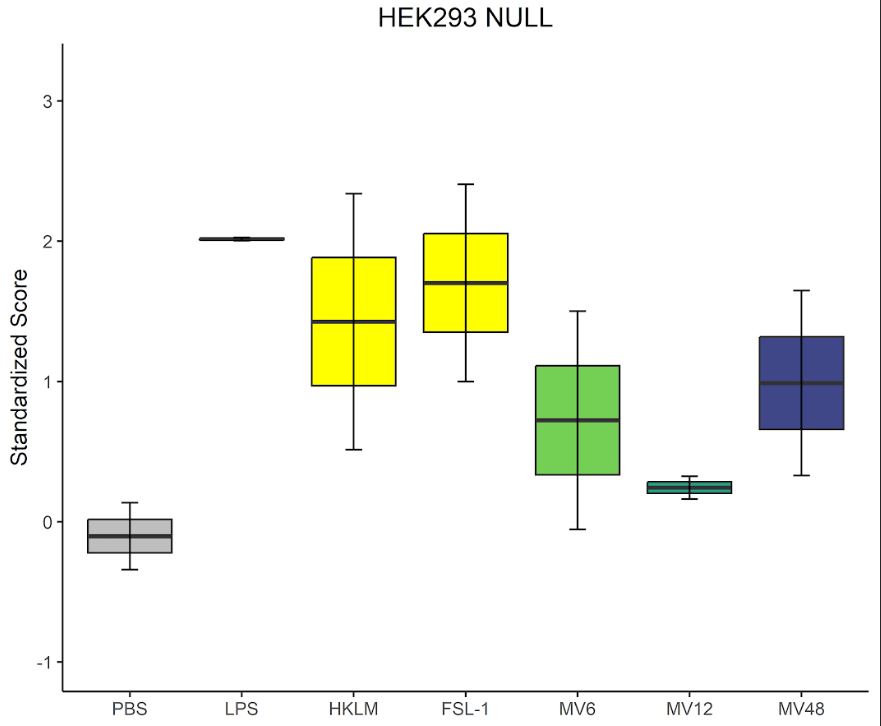


B
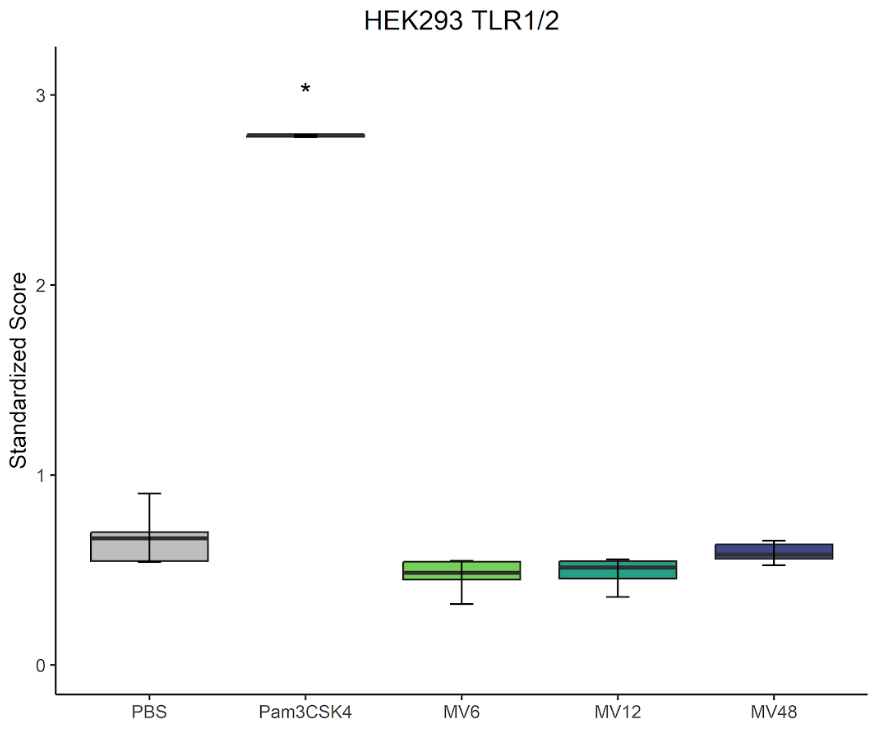


C
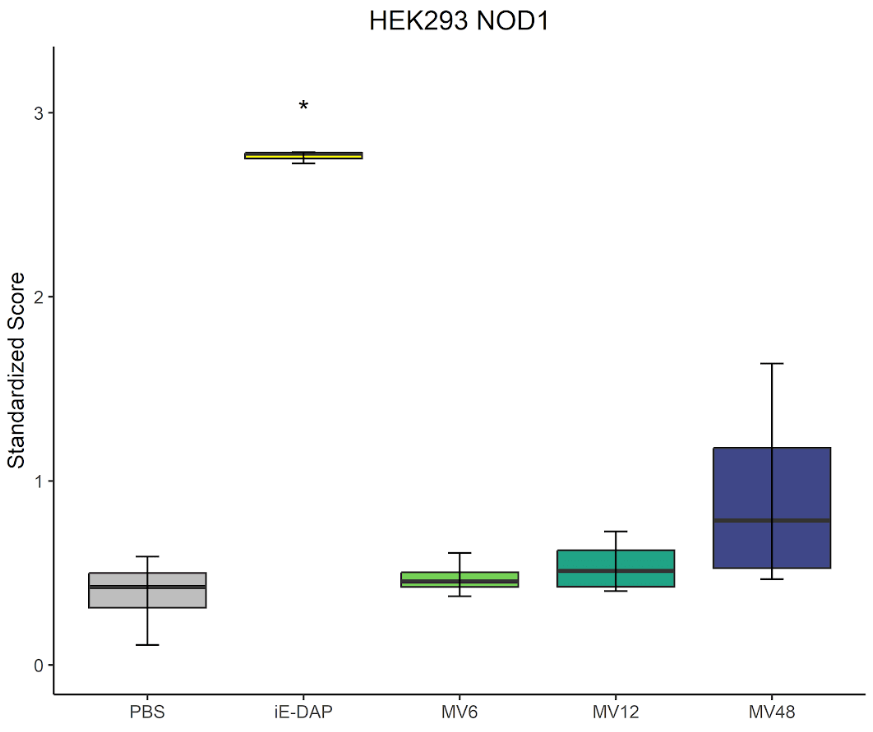


D
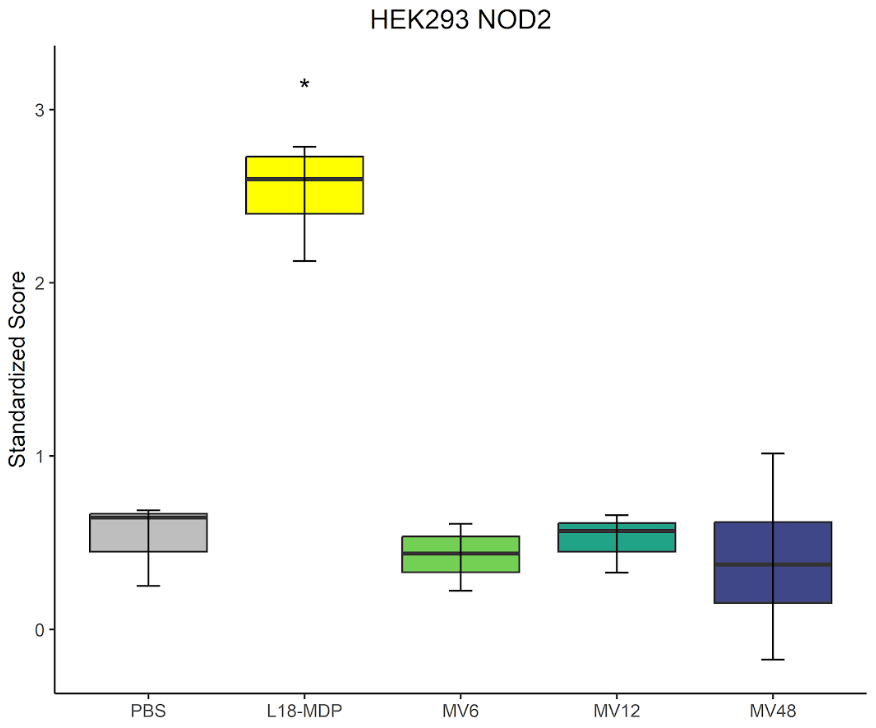


E
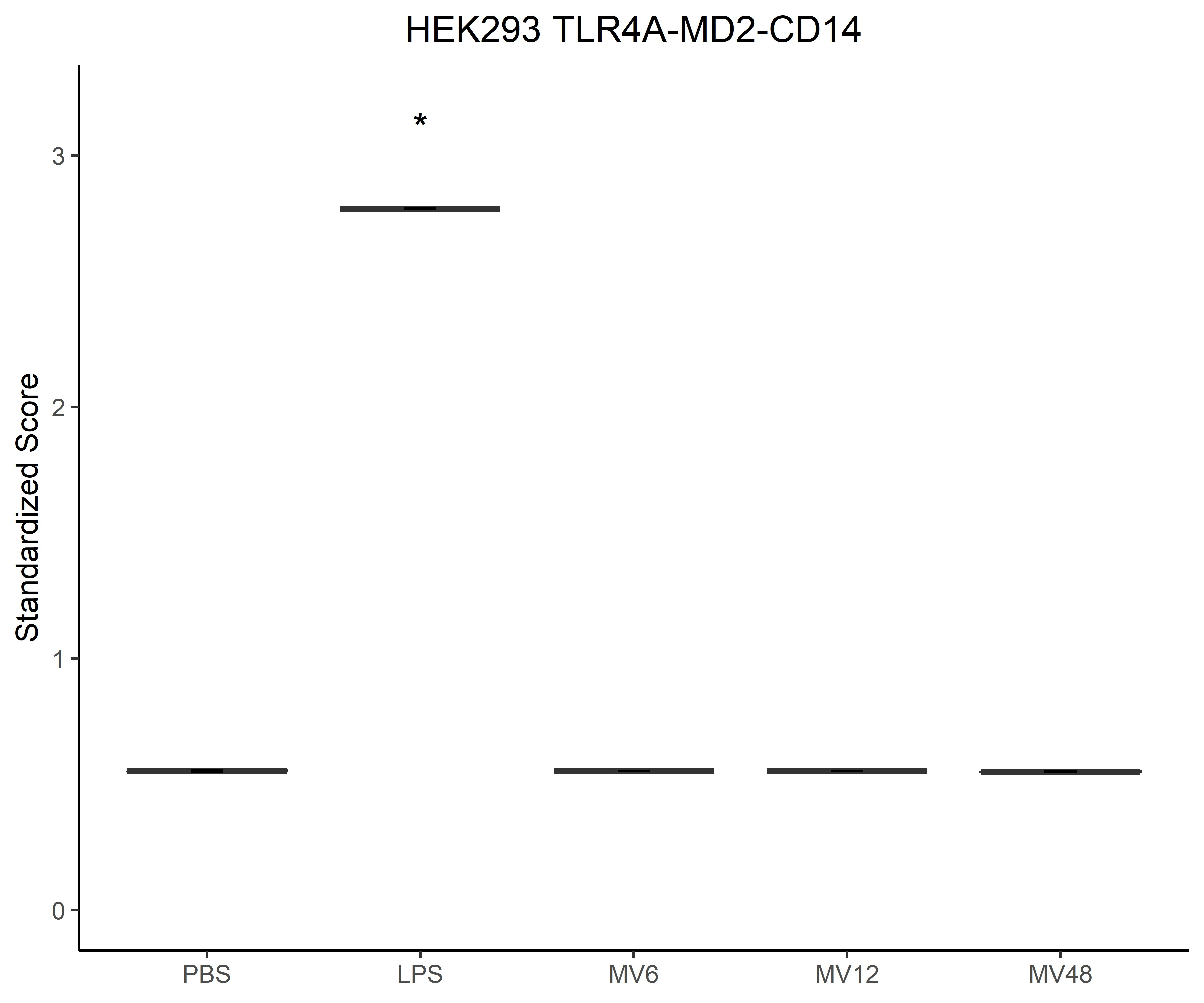


***Figure S8: Human cell receptors not involved in the recognition of membrane vesicles (MVs).*** *MVs isolated after 6, 12, and 48 h (MV6, MV12 and MV48) did not affect scrambled control (A) and activation of TLR1/2 (B), NOD1 (C), NOD2 (D), and TLR4A-MD2-CD14 (E), expressed on human embryonic kidney cells HEK-293 (concentration 10^5^ MVs per cell). The recognition abilities were compared to negative control (PBS), appropriate positive control is depicted (Pam3CSK4 for HEK293 TLR1/2, iE-DAP for HEK293 NOD1, L18-LDP for HEK293 NOD2, LPS for HEK293 TLR4A-MD2-CD14). Results were evaluated based on IL-8 production activity in cell culture media. The box plots show medians (lines), interquartile ranges (boxes), and minimum and maximum values (whiskers); data normalization was performed using Z-score; statistical analysis was performed using the ANOVA and the significance was assessed at * p < 0.05; n=4.*

***Table S1: Colony forming units per milliliter (CFUml^-1^).*** *Data are shown as mean ± SD; n=3.*


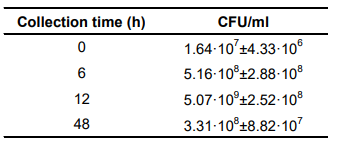


***Table S2: Kinetic parameters of Lacticaseibacillus rhamnosus-derived membrane vesicles (MVs) uptake by Caco-2 cells at 4 °C.*** *The parameters encompass the saturation plateau, kinetic rate constant (K), half-life (defined as the time required to attain half of the saturation level), and tau (signifying the time constant of saturation).*


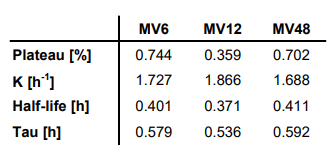


***Table S3: Protein localization prediction using PSORTb software.***

**
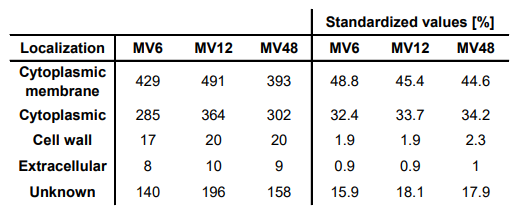
**

***Table S4: The number of proteins in each functional group annotated using KEGG Mapper****. The color scale indicates the ratio of protein representation in the individual groups (green – the largest, red – the smallest). The percentage values presented are relative to the total number of proteins.*

*
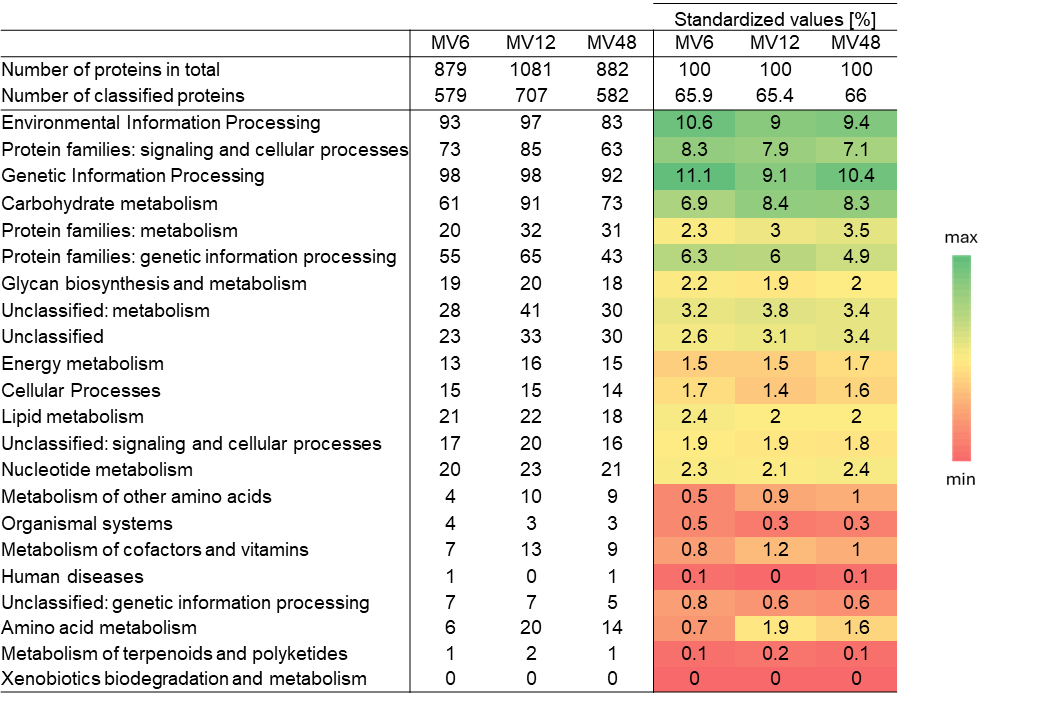
*

***Table S5: Ten most significantly up- and down-regulated proteins annotated in each combination of membrane vesicles (MVs)*** *(MV6-MV12, MV6-MV48, MV12-MV48).*

*
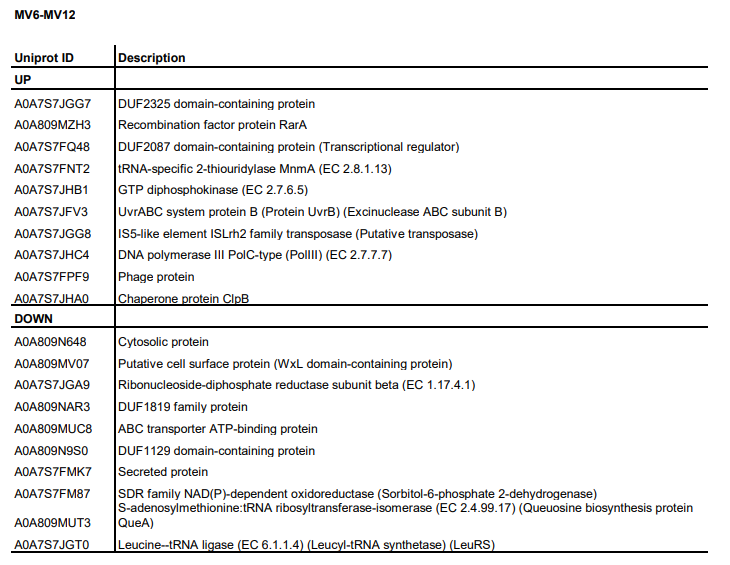
*

**
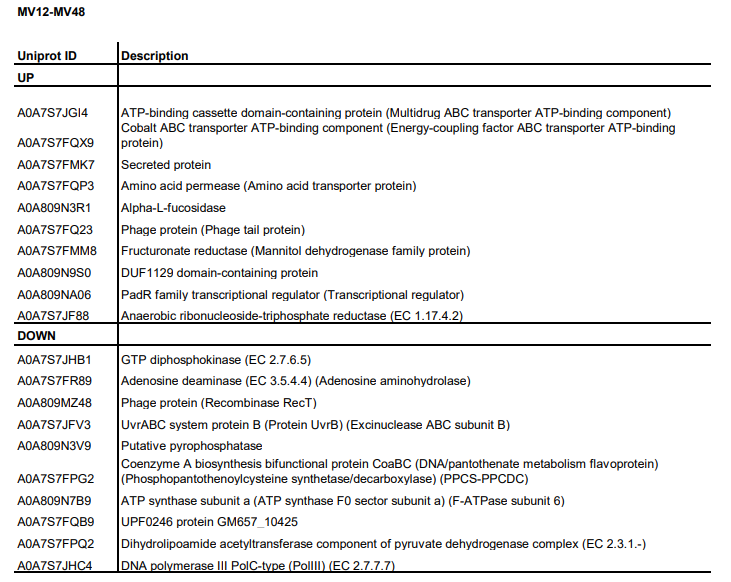
**

**
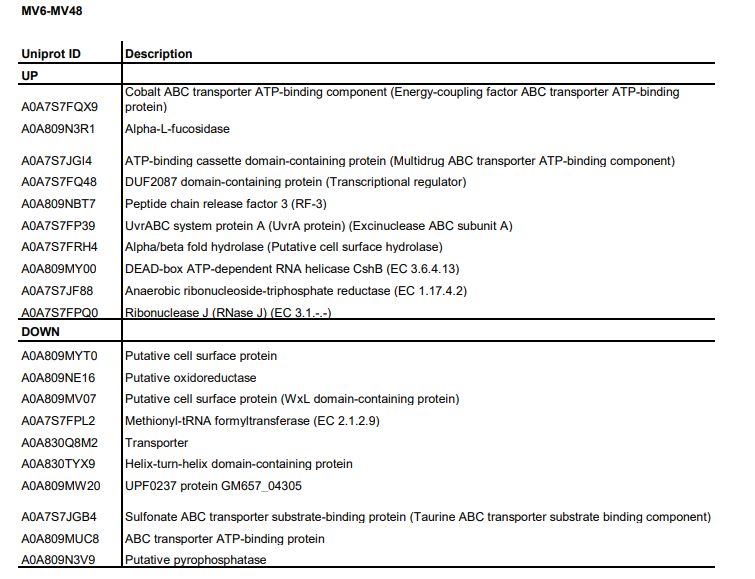
**

***Table S6: Predicted interaction network of upregulated proteins from Lacticaseibacillus rhamnosus CCM7091 membrane vesicles isolated throughout bacterial growth after 48 h (MV48) with murine TNFα signaling cascade.*** *Uniprot ID is used as a protein identifier for* *L.rhamnosus CCM7091 proteins (bacterial proteins). Uniprot name is used as a protein identifier for host protein. The interactions were predicted with host proteins, which were retrieved from the Reactome database [3]. The interolog prediction on the PredHPI Webserver [4] was employed to predict interactions, with the criteria set to a minimum coverage of 60% and a sequence identity threshold of 30% for each interacting partner.*

**
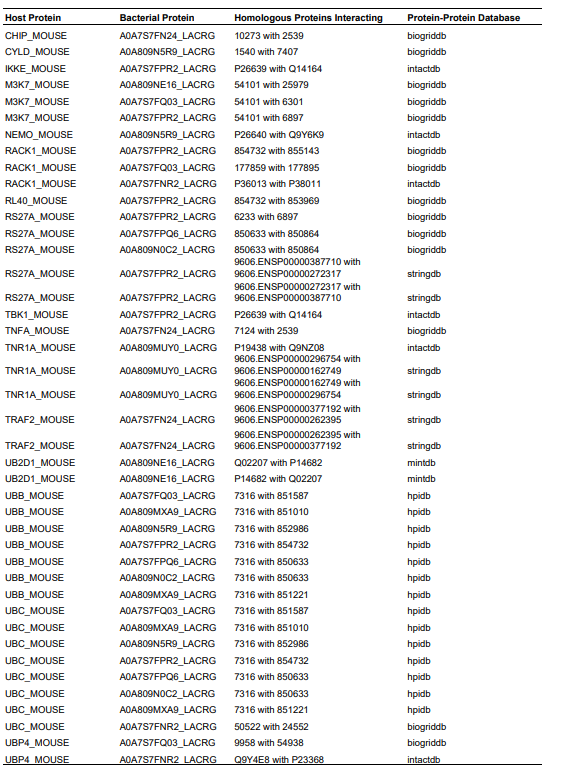
**

***Table S7: Predicted interaction network of upregulated proteins from Lacticaseibacillus rhamnosus CCM7091 membrane vesicles isolated throughout bacterial growth after 48 h (MV48) with murine IL-6 signaling cascade.*** *Uniprot ID is used as a protein identifier for* *L.rhamnosus CCM7091 proteins (bacterial proteins). Uniprot name is used as a protein identifier for host protein. The interactions were predicted with host proteins, which were retrieved from the Reactome database [3]. The interolog prediction on the PredHPI Webserver [4] was employed to predict interactions, with the criteria set to a minimum coverage of 60% and a sequence identity threshold of 30% for each interacting partner.*

**
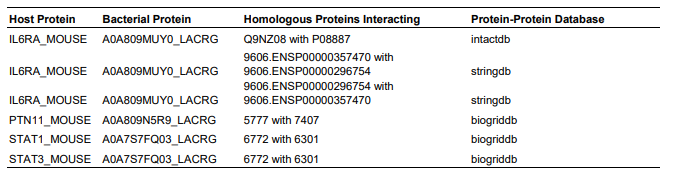
**

***Table S8: Predicted interaction network of upregulated proteins from Lacticaseibacillus rhamnosus CCM7091 membrane vesicles isolated throughout bacterial growth after 48 h (MV48) with murine IL-10 signaling cascade.*** *Uniprot ID is used as a protein identifier for* *L.rhamnosus CCM7091 proteins (bacterial proteins). Uniprot name is used as a protein identifier for host protein. The interactions were predicted with host proteins, which were retrieved from the Reactome database [3]. The interolog prediction on the PredHPI Webserver [4] was employed to predict interactions, with the criteria set to a minimum coverage of 60% and a sequence identity threshold of 30% for each interacting partner.*

***
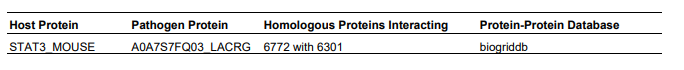
***

***Table S9: Predicted interaction network of upregulated proteins from Lacticaseibacillus rhamnosus CCM7091 membrane vesicles isolated throughout bacterial growth after 48 h (MV48) with human TLR cascade.*** *Uniprot ID is used as a protein identifier for* *L.rhamnosus CCM7091 proteins (bacterial proteins). Uniprot name is used as a protein identifier for host protein. The interactions were predicted with host proteins, which were retrieved from the Reactome database [3]. The interolog prediction on the PredHPI Webserver [4] was employed to predict interactions, with the criteria set to a minimum coverage of 60% and a sequence identity threshold of 30% for each interacting partner.*

***
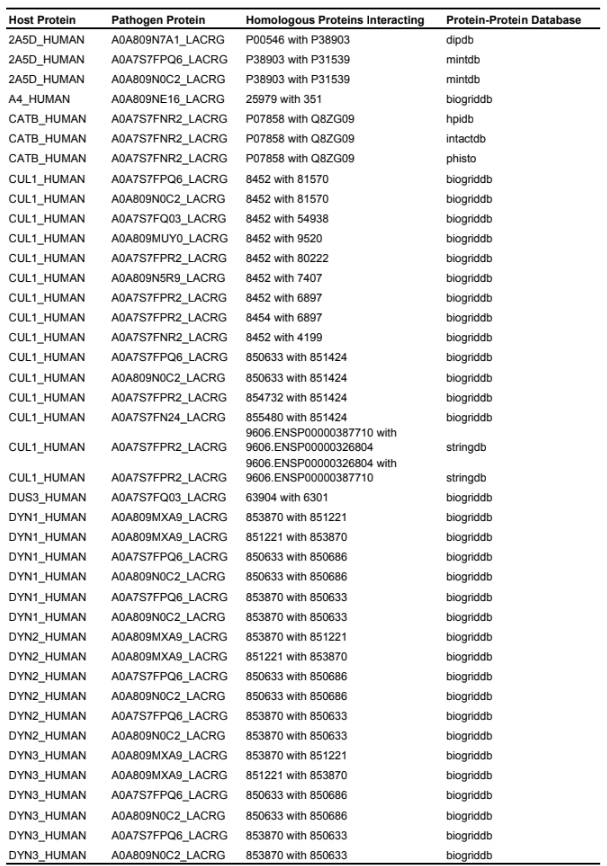
***

***
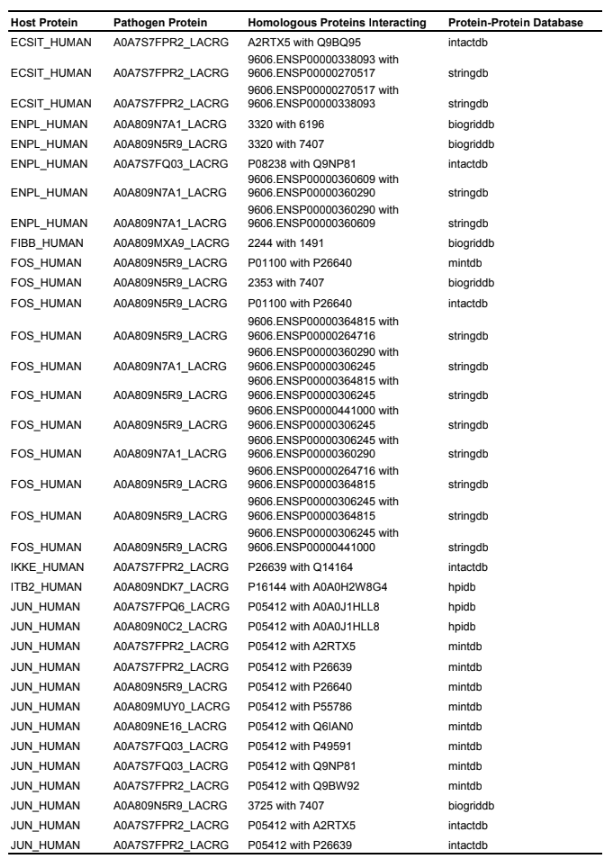
***

***
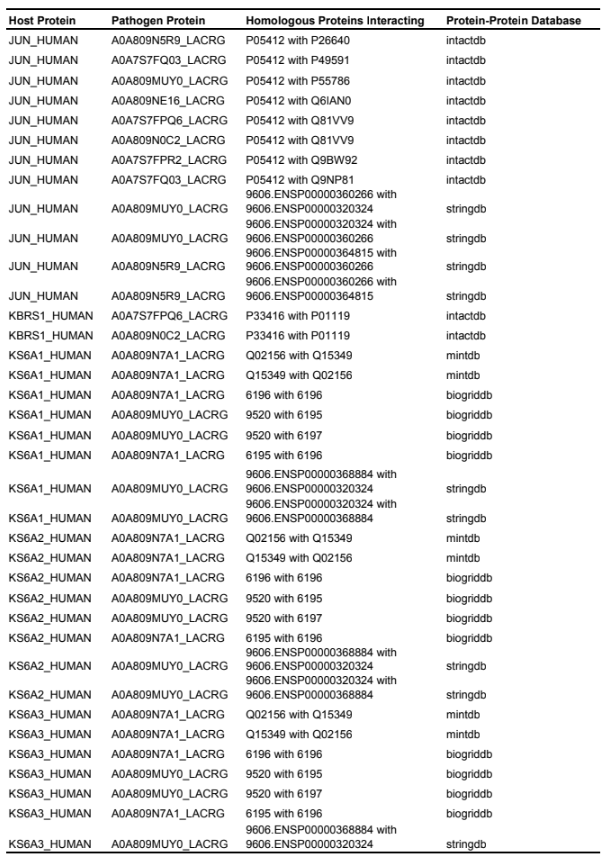
***

***
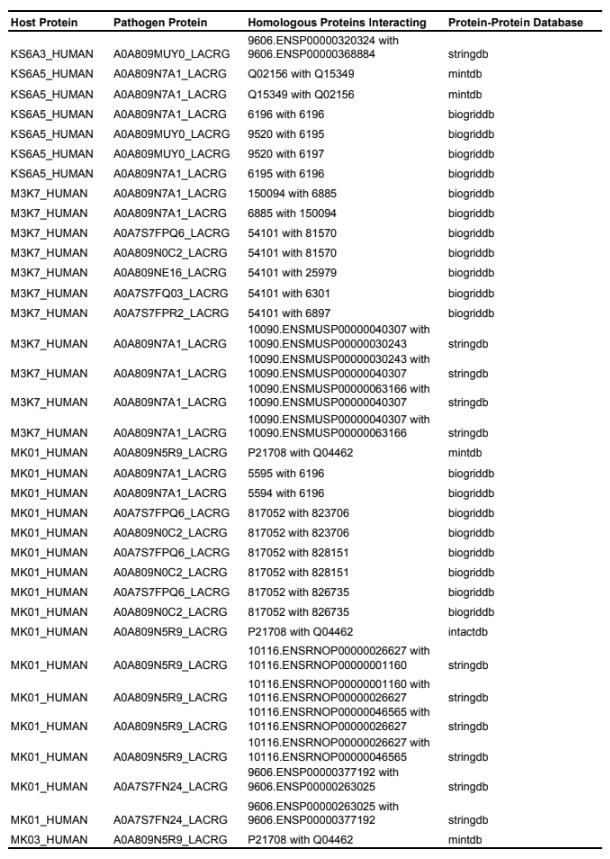
***

***
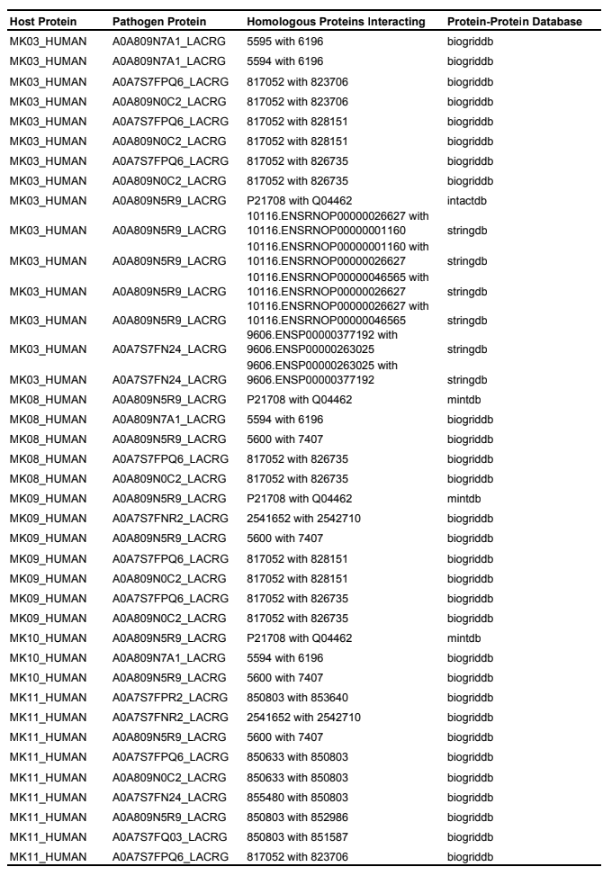
***

***
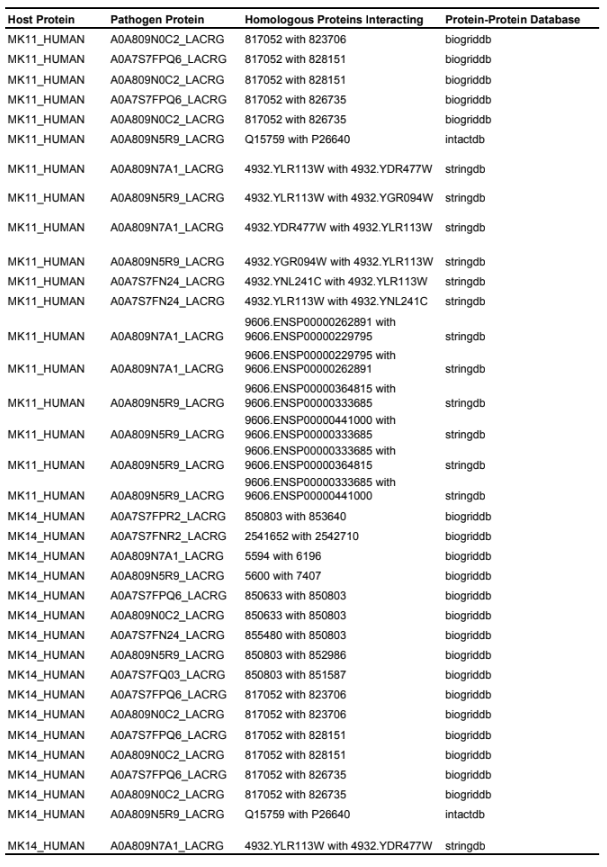
***

***
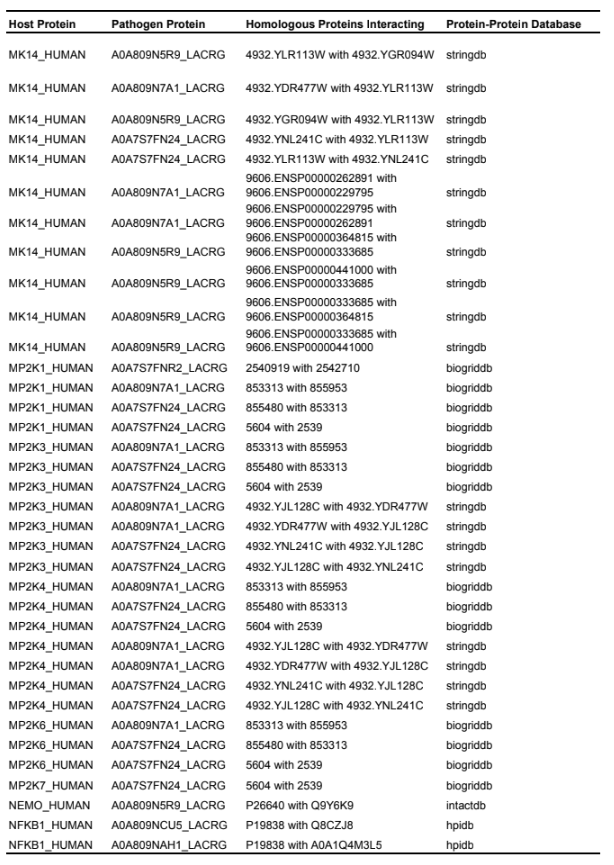
***

***
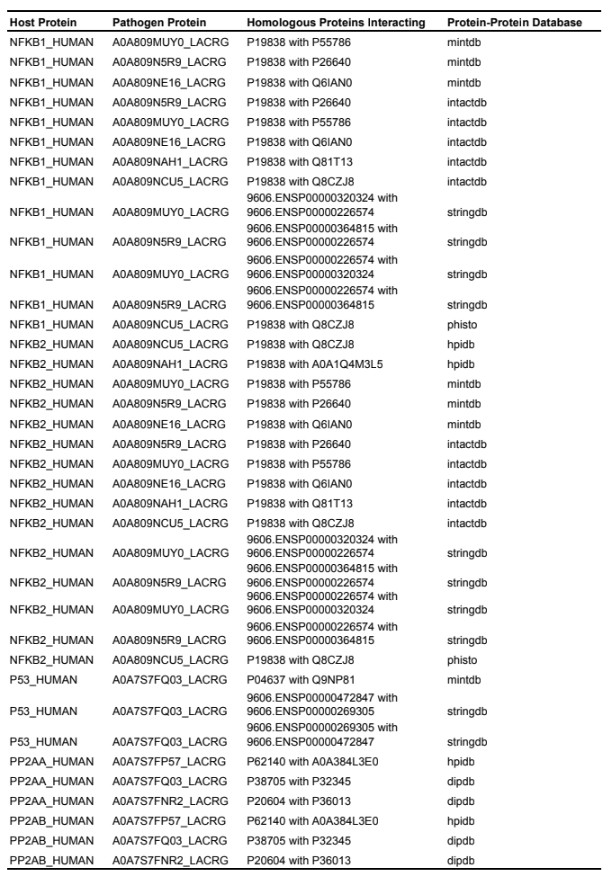
***

***
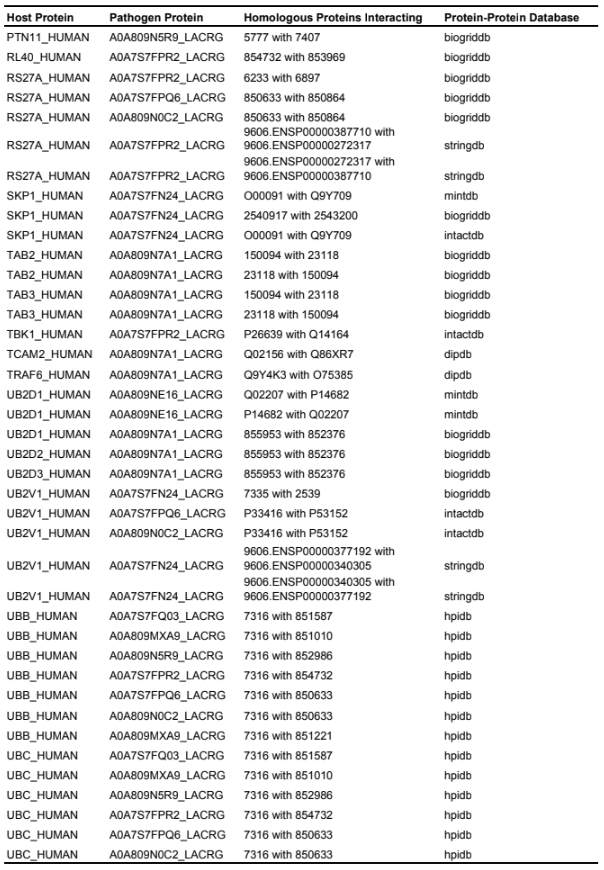
***

***
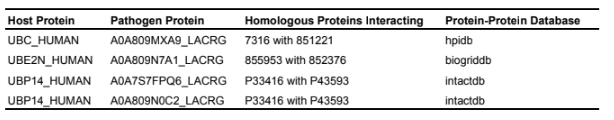
***

**References**

1. Wisniewski, J. R., Zougman, A., Nagaraj, N. & Mann, M. (2009) Universal sample preparation method for proteome analysis, *Nat Methods.* **6**, 359-62.

2. Demichev, V., Messner, C. B., Vernardis, S. I., Lilley, K. S. & Ralser, M. (2020) DIA-NN: neural networks and interference correction enable deep proteome coverage in high throughput, *Nat Methods.* **17**, 41-44.

3. Milacic, M., Beavers, D., Conley, P., Gong, C., Gillespie, M., Griss, J., Haw, R., Jassal, B., Matthews, L., May, B., Petryszak, R., Ragueneau, E., Rothfels, K., Sevilla, C., Shamovsky, V., Stephan, R., Tiwari, K., Varusai, T., Weiser, J., Wright, A., Wu, G., Stein, L., Hermjakob, H. & D'Eustachio, P. (2024) The Reactome Pathway Knowledgebase 2024, *Nucleic Acids Res.* **52**, D672-D678.

4. Loaiza, C. D. & Kaundal, R. (2021) PredHPI: an integrated web server platform for the detection and visualization of host-pathogen interactions using sequence-based methods, *Bioinformatics.* **37**, 622-624.
